# Podocyte-specific deletion of tubular sclerosis complex 2 promotes focal segmental glomerulosclerosis and progressive renal failure

**DOI:** 10.1371/journal.pone.0229397

**Published:** 2020-03-19

**Authors:** Wakiko Iwata, Hiroyuki Unoki-Kubota, Hideki Kato, Akira Shimizu, Michihiro Matsumoto, Toshiyuki Imasawa, Arisa Igarashi, Kenji Matsumoto, Tetsuo Noda, Yasuo Terauchi, Masaomi Nangaku, Masato Kasuga, Yasushi Kaburagi

**Affiliations:** 1 Department of Diabetic Complications, Diabetes Research Center, Research Institute, National Center for Global Health and Medicine, Tokyo, Japan; 2 Department of Endocrinology and Metabolism, Yokohama City University Graduate School of Medicine, Yokohama, Kanagawa, Japan; 3 Division of Nephrology and Endocrinology, Graduate School of Medicine, The University of Tokyo, Tokyo, Japan; 4 Department of Analytic Human Pathology, Nippon Medical School, Tokyo, Japan; 5 Department of Molecular Metabolic Regulation, Diabetes Research Center, Research Institute, National Center for Global Health and Medicine, Tokyo, Japan; 6 Kidney Center, National Hospital Organization Chiba-Higashi National Hospital, Chiba, Japan; 7 Department of Allergy and Clinical Immunology, National Research Institute for Child Health and Development, Tokyo, Japan; 8 Cancer Institute of Japanese Foundation for Cancer Research, Tokyo, Japan; 9 National Center for Global Health and Medicine, Tokyo, Japan; University of Utah School of Medicine, UNITED STATES

## Abstract

Obesity can initiate and accelerate the progression of kidney diseases. However, it remains unclear how obesity affects renal dysfunction. Here, we show that a newly generated podocyte-specific *tubular sclerosis complex 2 (Tsc2)* knockout mouse model (*Tsc2*^Δ*podocyte*^) develops proteinuria and dies due to end-stage renal dysfunction by 10 weeks of age. *Tsc2*^Δ*podocyte*^ mice exhibit an increased glomerular size and focal segmental glomerulosclerosis, including podocyte foot process effacement, mesangial sclerosis and proteinaceous casts. Podocytes isolated from *Tsc2*^Δ*podocyte*^ mice show *nuclear factor*, *erythroid derived 2*, *like 2*-mediated increased oxidative stress response on microarray analysis and their autophagic activity is lowered through the mammalian target of rapamycin (mTOR)—unc-51-like kinase 1 pathway. Rapamycin attenuated podocyte dysfunction and extends survival in *Tsc2*^Δ*podocyte*^ mice. Additionally, mTOR complex 1 (mTORC1) activity is increased in podocytes of renal biopsy specimens obtained from obese patients with chronic kidney disease. Our work shows that mTORC1 hyperactivation in podocytes leads to severe renal dysfunction and that inhibition of mTORC1 activity in podocytes could be a key therapeutic target for obesity-related kidney diseases.

## Introduction

The prevalence of obesity is increasing worldwide and contributes to many health problems, including type 2 diabetes mellitus (T2DM), cardiovascular disease and several types of cancer [1, 2]. Obesity, T2DM, hypertension and cardiovascular disease are all risk factors for chronic kidney disease (CKD) and end-stage renal disease [3, 4]. Several studies support the association between obesity and kidney disease. However, the precise mechanisms by which obesity contributes to the development and/or progression of CKD and end-stage renal disease are not completely understood. Some of the deleterious renal consequences of obesity may be mediated by inflammation induced by the production of cytokines and growth factors such as adiponectin, leptin and inflammatory cytokines [5].

Dysregulation of the mammalian target of rapamycin (mTOR) signalling pathway is also implicated in obesity [6]. mTOR, an evolutionarily conserved serine-threonine kinase, is part of a nutrient-sensing pathway regulating cellular growth, survival and metabolism. It interacts with several proteins to form two distinct complexes named mTOR complex 1 (mTORC1) and mTOR complex 2. In addition, mTORC1 is negatively regulated by a heterodimer complex containing tuberous sclerosis complex 1 (TSC1) and tuberous sclerosis complex 2 (TSC2) [7, 8]. mTORC1 is highly active in the tissues of obese and high-fat-fed-rodents [9]. In humans, the mTORC1 signalling effector S6K is upregulated in visceral fat tissues of obese patients [10]. Moreover, single nucleotide polymorphism analysis has revealed that a common genetic variation in regulatory-associated protein of mTOR (*RAPTOR)* is associated with overweight/obesity in American men of Japanese ancestry [11]. Inhibition of adipose mTORC1 signalling genetically impairs adipogenesis [12], whereas increased mTORC1 signalling promotes adipogenesis [13]. Recent reports have also shown that mTORC1 contributes to thermogenesis by modulating the brown-to-white adipocyte phenotypic switch [14, 15]. Accumulating evidence suggests that mTOR signalling could be a key regulator of obesity and its morbidities.

In this study, we hypothesize that mTORC1 activity might contribute to obesity-related renal functional decline. Accordingly, we generated podocyte-specific *Tsc2* knockout mice (*Tsc2*^Δ*podocyte*^), in which *Tsc2* is specifically depleted by *Podocin-Cre* [16]. Deletion of the *Tsc2* gene in podocytes increases glomerular size and the characteristics of focal segmental glomerulosclerosis (FSGS) and causes end-stage renal dysfunction concomitant with impaired autophagy in podocytes. Assessment of the involvement of mTORC1 in human kidney biopsy specimens demonstrated that mTORC1 signalling was surprisingly activated in podocytes from obese patients with CKD.

## Materials and methods

### Animals

Male obese *db/db* mice and their nonobese controls (*db/m*) were obtained from CLEA Japan Inc. (Tokyo, Japan). Mice with exons 3 and 4 of the *Tsc2* gene flanked by two *lox P* sequences have previously been reported [17]. Heterozygous *Nphs2-Cre* transgenic mice were provided by Dr. Susan E Quaggin (Feinberg Cardiovascular Research Institute, Northwestern University, Chicago, IL) [16]. For generation of homozygous floxed *Tsc2* mice on an ICR background, which are more sensitive to glomerular diseases, mice were backcrossed for more than ten generations (Jcl:ICR; CLEA Japan Inc.). Offspring from the backcrossed *Tsc2*^*flox/flox*^ mice were crossed with *Nphs2-Cre* mice to generate mice heterozygous for the *Tsc2*-floxed allele (genotype: *Tsc2*^*flox/wt*^, *Nphs2-Cre*^+/-^). These mice were bred with *Tsc2*^*flox/flox*^ mice to inactivate both *Tsc2* alleles by Cre-mediated excision, thereby creating conditional knockout mice in which the *Tsc2* gene was specifically disrupted in podocytes (*Tsc2*^Δ*podocyte*^, genotype: *Tsc2*^*flox/flox*^, *Nphs2-Cre*^+/-^). PCR was used for *Tsc2 loxP* and *Nphs2-Cre* genotyping. *Tsc2*^*flox/flox*^ (genotype: *Tsc2*^*flox/flox*^, *Nphs2-Cre*^-/-^) and *Nphs2-Cre* (genotype: *Tsc2*^*wt/wt*^, *Nphs2-Cre*^+/-^) littermates were used as controls.

During the study, animals were housed in a temperature-controlled room (22°C) with a 12-h light/dark cycle with free access to diet and water. A standard laboratory diet (Labo H Standard, Nosan Corporation, Yokohama, Japan) was administered *ad libitum* from weaning. All animal care and procedures were performed in accordance with Animal Research Reporting In Vivo Experiments guidelines [18]. All research staff handling with animals was trained in accordance with the recommendations of the Institutional Animal Care and Use Committee of National Center for Global Heal and Medicine.

### Survival time

For survival analysis, at least ten *Tsc2*^Δ*podocyte*^ mice were followed open end for max. The survival time of *Nphs2-Cre* (*n* = 9), *Tsc2*^*flox/flox*^ (*n* = 10), and *Tsc2*^Δ*podocyte*^ (*n* = 32) mice was checked until 16 weeks of age and evaluated using the Kaplan-Meier method. For rapamycin treatment, *Nphs2-Cre* (*n* = 17), *Tsc2*^*flox/flox*^ (*n* = 16), and *Tsc2*^Δ*podocyte*^ (*n* = 8) mice were intraperitoneally injected with rapamycin (LC Laboratories, Woburn, MA) at 2 mg kg^-1^ of body weight every other day from 4 to 11 weeks of age. Saline injection for control was performed similarly (*Nphs2-Cre* [*n* = 7], *Tsc2*^*flox/flox*^ [*n* = 9], and *Tsc2*^Δ*podocyte*^ [*n* = 26]). All mice were monitored every 2 weeks beginning at 3 weeks of age. As described later, *Tsc2*^Δ*podocyte*^ mice had significantly shorter survival than control mice without humane intervention due to renal dysfunction. When mice exhibited reduced locomotor activity and hypothermia, blood urea nitrogen (BUN) was measured using an Arkray Spotchem D (Arkray, Kyoto, Japan). In case of BUN over 50 mg/dL, as a specific endpoint criterion, the affected mice were euthanized immediately. There were no mice that were euthanized before reaching the experimental endpoint. The numbers of mice that died without humane intervention and euthanized after reaching the experimental endpoint were summarized in S1 Table.

### Serum and urine analysis

At 3, 5 and 7 weeks of age, mice were individually placed in metabolic cages (Shinano Manufacturing, Tokyo, Japan) with free access to diet and water, and urine was collected for 16 h. Urinary albumin and creatinine levels were measured on a Hitachi 7180 analyser (Hitachi Inc., Tokyo Japan), and the albumin-to-creatinine ratio (ACR) was calculated. Body weight and fasting plasma glucose levels were measured, and blood samples were obtained as described previously [19]. Serum creatinine (SCr), total protein, albumin, uric acid, BUN, HDL-cholesterol (HDL-C), total cholesterol (TC), triglyceride, Na, K and Cl were measured using an Arkray Spotchem D (Arkray, Kyoto, Japan).

### Histological assessments

At defined experimental time points, mice were deeply anesthetized with sevoflurane (Maruishi Pharmaceutical Co., Ltd, Osaka, Japan). The sacrificed mice were perfused with 0.9% NaCl solution and then both kidneys were excised. Kidneys were fixed in 10% phosphate-buffered formalin, embedded in paraffin and deparaffinized in xylene; then 2-μm sections were stained with periodic acid-Schiff (PAS) and Masson’s trichrome. Glomerulosclerotic injury was graded based on the severity of glomerular damage, essentially as reported previously [20]. A glomerulosclerotic index was then calculated using the formula: Glomerulosclerotic index = (1 × n_1_) + (2 × n_2_) + (3 × n_3_) + (4 × n_4_) / (n_0_ + n_1_ + n_2_ + n_3_ + n_4_), where n_x_ is the number of glomeruli at each grade of glomerulosclerosis. At least 50 glomerular sections were randomly assessed in each mouse (*n* = 3/genotype), and this analysis was performed with the observer masked to the treatment groups. For an evaluation of glomerular size, glomerular diameters were assessed in 20 glomerular sections that were randomly selected from each mouse (*n* = 3/genotype), measured by using ImageJ processing software version 1.50i [21], and the averages of the glomerular diameters per glomerular section were calculated. For immunofluorescence studies, 4-μm frozen sections of OCT-embedded frozen kidneys were fixed in ice-cold acetone, blocked with 3% bovine serum albumin and incubated with primary antibodies—rabbit anti-Wilms tumor 1 (WT1) (1:50, sc-192, Santa Cruz Biotechnology, Dallas, TX), anti-synaptopodin (1:50, sc-50459, Santa Cruz Biotechnology) and anti-podocin (1:100, P0372, Sigma-Aldrich, St. Louis, MO) polyclonal antibodies and developed using FITC-conjugated swine anti-rabbit immunoglobulins polyclonal antibody (1:20, F020502, Dako; Agilent Technologies, Santa Clara, CA). Cell nuclei were counterstained with Hoechst 33342 and mounted with Fluoromount. The numbers of double-positive cells (WT1 and Hoechst 33342) were counted in more than 20 glomerular sections that were randomly selected from each mouse (*n* = 6–8/genotype) and the averages of the double-positive cells per glomerular section were calculated.

### Transmission electron microscopic analysis

Kidney samples were fixed with 2.5% glutaraldehyde in phosphate buffer (pH 7.4), postfixed with 1% osmium tetroxide, dehydrated, and embedded in Epok 812. Ultrathin sections were stained with uranyl acetate and lead citrate and then examined with a transmission electron microscope (H-7100, Hitachi Ltd., Tokyo, Japan). Glomerular basement membrane thickness was assessed in 22–25 fields in the glomeruli, which were randomly selected from each mouse, and was measured by using ImageJ processing software version 1.50i [21].

### Isolation of glomeruli and culture of primary podocytes

Glomeruli of *Tsc2*^Δ*podocyte*^ and control mice were isolated by magnetic bead isolation [22]. Isolated glomeruli were cultured on type I collagen-coated multiwell plate dishes (AGC Techno Glass Co. Ltd., Shizuoka, Japan) in RPMI 1640 (Wako Pure Chemical Industries, Ltd., Osaka, Japan) containing 10% fetal bovine serum (GE Healthcare, Chicago, IL) supplemented with 100 U ml^-1^ penicillin and 100 μg ml^-1^ streptomycin (Thermo Fisher Scientific, Inc., Waltham, MA) in a 37°C humidified incubator with 5% CO_2_. Explant primary podocytes were used for subsequent analyses. The podocytes isolated from these mice were stained with WT1, a podocyte marker, and the ratio of the number of WT1-positive cells to the number of the explant cells was 96.3 ± 2.1%. For an LC3B assay, the cultured podocytes were treated with or without 10 μM chloroquine for 24 h before analysis.

### RNA extraction and quantitative real-time PCR

Total RNA was isolated using an RNeasy Mini kit (Qiagen, Hilden, Germany), and cDNA was synthesized using ReverTra Ace qPCR RT Master Mix (Toyobo, Osaka, Japan). Fast SYBR Green and TaqMan Fast Advantage (Thermo Fisher Scientific) were used for real-time PCR analysis and the expression levels of each mRNA were quantified using the standard curve method and normalized relative to the levels of expression of β-actin or GAPDH mRNA in the same sample.

### Microarray analysis

Total RNA isolated from the podocytes of *Tsc2*^Δ*podocyte*^ and *Tsc2*^*flox/flox*^ mice was subjected to microarray analysis. RNA quality and integrity were determined using the Agilent RNA 6000 Nano Kit on the Agilent 2100 Bioanalyzer (Agilent Technologies, Böblingen, Germany). All samples were analysed with Agilent SurePrint G3 Mouse GE 8×60K microarray (Agilent Technologies). Sample labelling, microarray hybridization and washing were performed according to the manufacturer’s instructions using the One-Color Microarray-Based Gene Expression Analysis Protocol. Data extraction was performed using Feature Extraction Software, and the Feature Extraction Software-derived output data files were further analysed using GeneSpring software (version 14.8, Agilent Technologies). Differentially expressed mRNAs were selected on the basis of a fold-change ≥ |1.5| at *P* < 0.05 between the *Tsc2*^Δ*podocyte*^ mice and control samples by the Benjamini-Hochberg procedure. To facilitate gene microarray data analysis, Ingenuity Pathway Analysis software (Qiagen, Redwood City, CA) was used for *in silico* genomics network analysis to search for possible biological processes, pathways and networks.

### Immunoblot analysis

Cultured podocytes were lysed in RIPA buffer containing phosphatase inhibitor (Nacalai Tesque, Kyoto, Japan). The samples were resolved by 7.5% or 10–20% SDS-PAGE and transferred to Immobilon-P Transfer Membranes (Merck Millipore, Billerica, MA). The membranes were incubated with the antibodies indicated below and washed and incubated with secondary antibodies. Blots were visualized using an enhanced chemiluminescence system (Amersham ECL Prime Western Blotting Detection Reagent, GE Healthcare). Images were captured with a ChemiDoc XRS+ system and processed using ImageLab software (Bio-Rad Laboratories, Hercules, CA). Antibodies used in this study were as follows: rabbit anti-tuberin (1:1000, sc-893, Santa Cruz Biotechnology), anti-WT1 (1:500, sc-192, Santa Cruz Biotechnology), anti-p62 (1:5000, P0067, Sigma-Aldrich), anti-LC3B (1:5000, L7543, Sigma-Aldrich), anti-phosphorylated unc-51-like kinase 1 (ULK1) (Ser757) (1:1000, #6888, Cell Signaling Technology, Beverly, MA), anti-phosphorylated p70S6K (Thr389) (1:1000, #9205, Cell Signaling Technology), anti-phosphorylated eukaryotic translation initiation factor 4E- binding protein 1 (4EBP1) (Ser65) (1:1000, #9451, Cell Signaling Technology) polyclonal antibodies and mouse anti-β-tubulin monoclonal antibody (1:1000, 05–661, Upstate Biotechnology, Lake Placid, NY).

### Quantitative analysis of autophagic activity *in vivo*

GFP-LC3 transgenic mice provided by N. Mizushima (The University of Tokyo, Tokyo, Japan) were used to analyse autophagic activity *in vivo* [23]. *Tsc2*^Δ*podocyte*^ and control mice were subsequently crossed with GFP-LC3 transgenic mice. The numbers of GFP-LC3 puncta in podocytes were observed with an LSM880 confocal microscope (Zeiss, Oberkochen, Germany), counted in 20 glomeruli randomly selected from each mouse (*n* = 3/genotype) and quantified.

### Human kidney biopsy specimens

Clinically indicated renal biopsies were performed in obese patients (body mass index > 25 kg m^-2^) with CKD at Chiba-Higashi National Hospital. Three human kidney biopsy specimens from patients diagnosed with FSGS perihilar variant were stained with rabbit anti-phospho-S6 ribosomal protein (pS6) monoclonal antibody (Ser235/236, 1:400, #4858, Cell Signaling Technology) and counterstained with haematoxylin. pS6-positive glomeruli were counted, and the percentage of pS6-positive glomeruli in 10–26 glomeruli was calculated for each patient. Human kidney biopsy specimens obtained from three patients with abnormal results on urinalysis but with no glomerular abnormality on kidney biopsy were used as normal controls.

### Statistics

Kaplan-Meier analysis was conducted using IBM SPSS software version 20. Data are expressed as means ± s.d. for normally distributed variables and median (interquartile range) for non-normally distributed variables. Differences between the two groups for normally distributed variables were tested using Student’s two-sided *t*-test, and nonparametric data were analysed using the Mann–Whitney *U*-test. Differences among more than three groups were analysed using parametric (one-way analysis of variance) or nonparametric (Kruskal-Wallis test) statistical methods. All calculations were performed with Microsoft Excel 2016 or IBM SPSS software version 20. *P* < 0.05 was considered significant.

### Study approval

All animal protocols and experiments were approved by the Institutional Animal Care and Use Committee of National Center for Global Heal and Medicine (no. 18068). Human renal biopsies were performed at Chiba-Higashi National Hospital after written informed consent was received from participants prior to inclusion in the study. The protocol concerning the use of biopsy samples was approved by the ethics committee of Chiba-Higashi National Hospital (no. 27–14).

## Results

### *Tsc2* deletion in podocytes causes death due to renal failure

**Fig 1:**
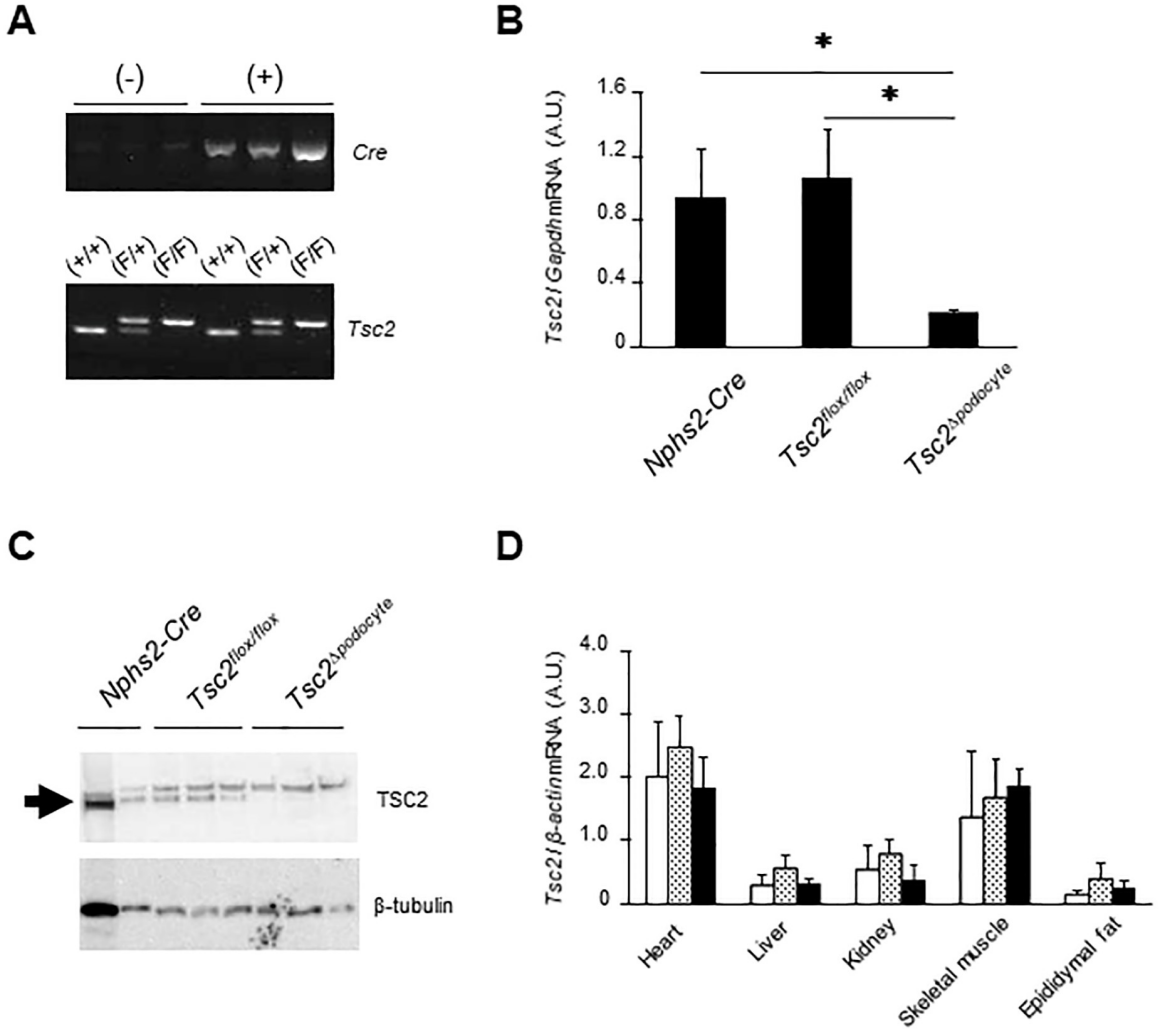
Generation of podocyte-specific *Tsc2* knockout mice, *Tsc2*^Δ*podocyte*^. Homozygous floxed *Tsc2* mice were crossed with *Nphs2-Cre* transgenic mice to generate *Tsc2*^Δ*podocyte*^, *Nphs2-Cre*^+/-^; *Tsc2*^*flox/flox*^. (**A**) PCR genotyping of genomic DNA from mouse tails. *NPHS2-Cre*^+/-^, *Tsc2*^*wt/wt*^ mice (*Nphs2-Cre*) and *NPHS2-Cre*^-/-^, *Tsc2*^*flox/flox*^ mice (*Tsc2*^*flox/flox*^) were used as controls. F, flox; +, wt. (**B**) Primary cultured podocytes isolated from *Tsc2*^Δ*podocyte*^ and wild-type control mice at 4 weeks of age demonstrated that *Tsc2* mRNA was knocked down by over 80% in the podocytes of *Tsc2*^Δ*podocyte*^ mice compared with control mice. Results are expressed as means ± s.d. *P < 0.05 compared with age-matched controls. (**C**) Western blot analysis of primary cultured podocytes isolated from *Tsc2*^Δ*podocyte*^ and control mice. TSC2 gives a band at 200 kDa (*arrow*). Non-specific bands were also detected above the TSC2 band in all samples. β-Tubulin was used as an internal control. (**D**) *Tsc2* mRNA expression in various tissues of *Tsc2*^Δ*podocyte*^ and wild-type controls. Expression levels of *Tsc2* mRNA in *Tsc2*^Δ*podocyte*^ were comparable with those in controls in all the tissues examined, including the renal cortex. White bar, *Nphs2-Cre*; dotted bar, *Tsc2*^*flox/flox*^; black bar, *Tsc2*^Δ*podocyte*^.

We generated *Tsc2*^Δ*podocyte*^ mice by crossing homozygous floxed *Tsc2* mice (*Tsc2*^*flox/flox*^) with Cre-recombinase transgenic mice that had *Cre* gene under the control of a murine *Podocin* (*Nphs2*) promoter (*Nphs2-Cre*). *Tsc2*^Δ*podocyte*^ mice were born at the expected Mendelian ratio and divided into three types according to genotype: *Nphs2-Cre*, *Tsc2*^*flox/flox*^ and *Tsc2*^Δ*podocyte*^ (Fig 1A). To verify the depletion of *Tsc2* in podocytes, we examined mRNA from primary cultured podocytes. The *Tsc2* mRNA level showed an 80% ± 0.42% reduction in *Tsc2*^Δ*podocyte*^ mice compared with control mice (Fig 1B), and TSC2 protein was barely detected in *Tsc2*^Δ*podocyte*^ mice. (Fig 1C). We also examined the tissue distribution of *Tsc2* mRNA, including the renal cortex; however, *Tsc2* mRNA levels did not differ in the tissues examined between *Tsc2*^Δ*podocyte*^ mice and control mice (Fig 1D).

**Fig 2:**
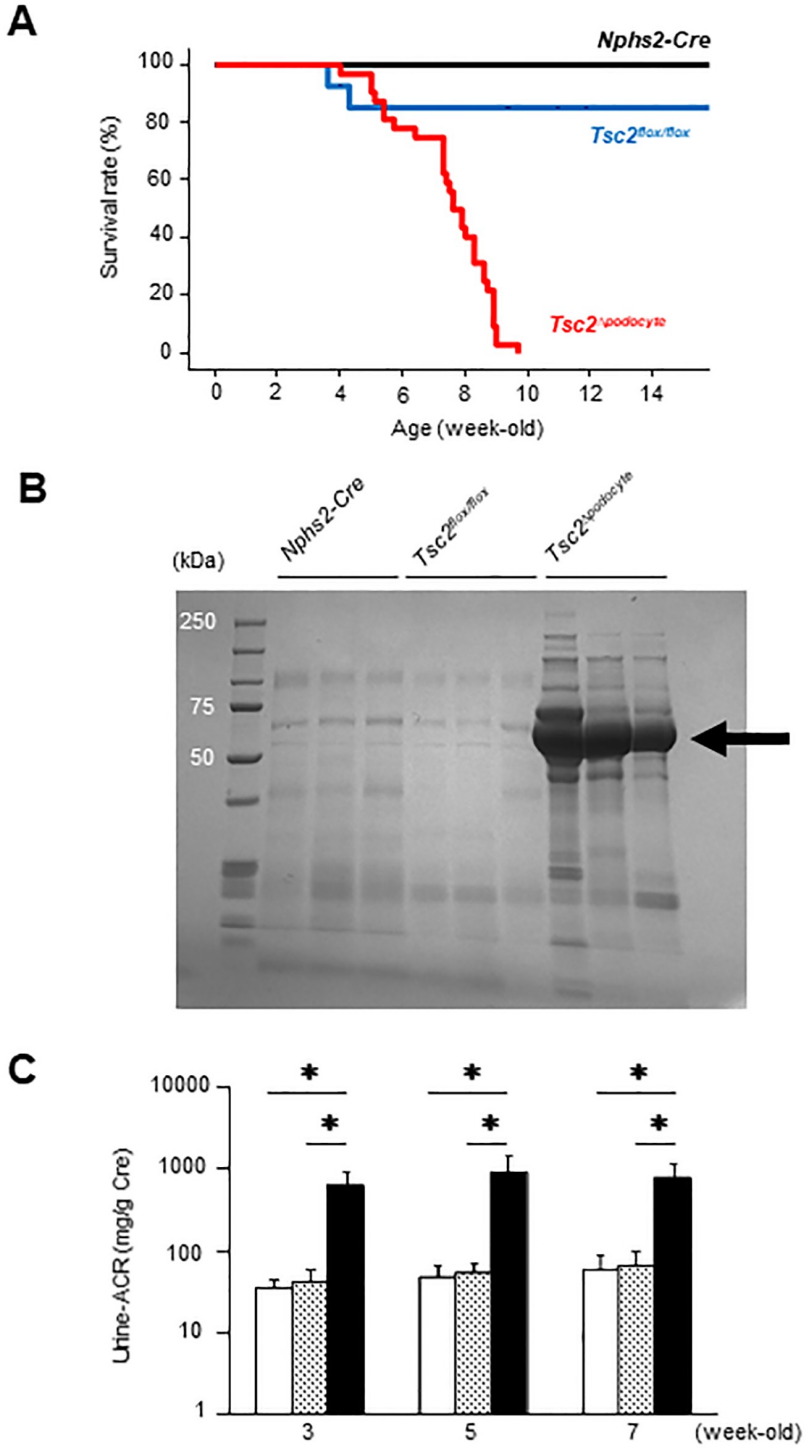
mTORC1 activation in podocytes causes proteinuria and increased mortality. (**A**) Kaplan-Meier survival plots for *Tsc2*^Δ*podocyte*^ and control mice. A significant increase in mortality was found in *Tsc2*^Δ*podocyte*^ compared with control mice. *Nphs2-Cre* (*black line*), *n* = 9; *Tsc2*^*flox/flox*^ (*blue line*), *n* = 10; *Tsc2*^Δ*podocyte*^ (*red line*), *n* = 32. (**B**) At 3 weeks, *Tsc2*^Δ*podocyte*^ became albuminuric. SDS-PAGE gel shows a microliter of urine was loaded for each lane. (**C**) Seven-week follow-up of *Tsc2*^Δ*podocyte*^ for proteinuria (*n* = 10 per group). Results are expressed as means ± s.d. White bar, *Nphs2-Cre*; dotted bar, *Tsc2*^*flox/flox*^; black bar, *Tsc2*^Δ*podocyte*^. Results are expressed as means ± s.d. *P < 0.05 compared with age-matched controls.

*Tsc2*^Δ*podocyte*^ mice were normoglycemic and nonobese, and initially appeared normal. However, Kaplan-Meier analysis indicated that *Tsc2*^Δ*podocyte*^ mice had significantly shorter survival (*P* < 0.01) than control mice (Fig 2A). A dramatic loss of animals was detected in *Tsc2*^Δ*podocyte*^ mice after 4 weeks of age, and all of the *Tsc2*^Δ*podocyte*^ mice examined died by 10 weeks of age. SDS-PAGE analysis revealed that *Tsc2*^Δ*podocyte*^ mice started to develop albuminuria at approximately 3 weeks of age (Fig 2B). The urinary ACR also remained significantly higher in *Tsc2*^Δ*podocyte*^ mice than in control mice up to 7 weeks of age (Fig 2C). The levels of serum albumin and total protein in *Tsc2*^Δ*podocyte*^ mice began to decrease at 5 weeks of age. On the other hand, the levels of BUN and SCr began to increase in *Tsc2*^Δ*podocyte*^ mice (S2 Table). We found increased levels of K, TC, triglyceride and HDL-C in *Tsc2*^Δ*podocyte*^ mice at 7 weeks of age (S2 Table), and also observed massive ascites in *Tsc2*^Δ*podocyte*^ mice. There were few sex differences in the biochemical parameters examined (S2 Table). These findings indicate that specific deletion of *Tsc2* in podocytes led to death from renal dysfunction.

We further generated *Tsc2*^Δ*podocyte*^ mice of the C57BL/6 strain and found that these mice showed kidney dysfunction such as proteinuria and hypoalbuminemia and increased levels of TC and HDL-C (S1A–S1D Fig) at 24 weeks of age. However, the levels of BUN and SCr in *Tsc2*^Δ*podocyte*^ mice were comparable to those of the control mice (BUN in *Tsc2*^*flox/flox*^: 43.2 ± 8.8 mg/dL, BUN in *Tsc2*^Δ*podocyte*^: 47 ± 9.6 mg/dL; SCr in *Tsc2*^*flox/flox*^: 0.32 ± 0.14 mg/dL, SCr in *Tsc2*^Δ*podocyte*^: 0.14 ± 0.06 mg/dL). Kaplan-Meier analysis also revealed that these mice showed the same trend as the ICR strain (S1E Fig).

### *Tsc2*^Δ*podocyte*^ showed an increased glomerular size and FSGS

**Fig 3:**
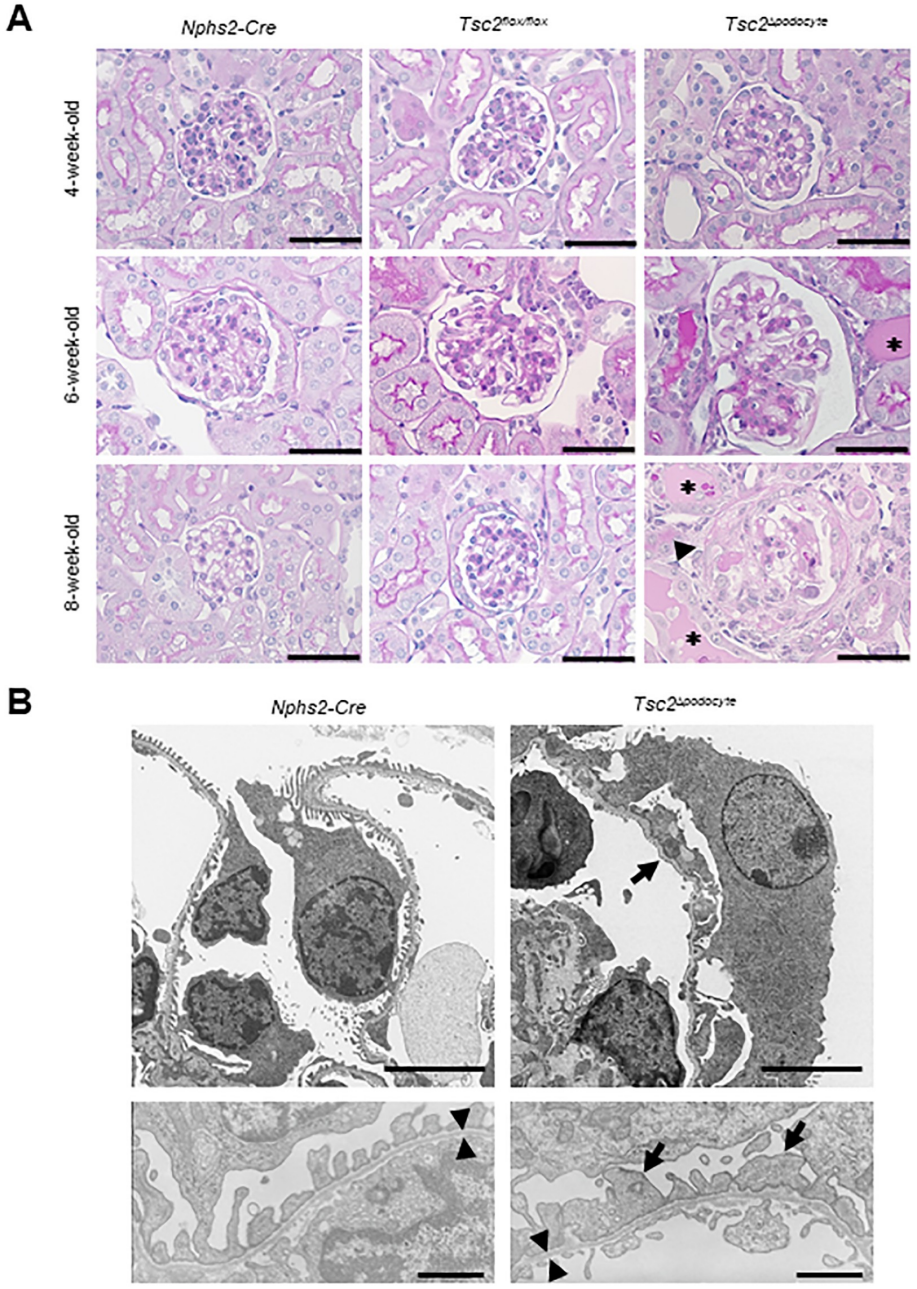
mTORC1 activation in podocytes resulted in progressive glomerulosclerosis. (**A**) *Tsc2*^Δ*podocyte*^ developed progressive glomerulosclerosis between 4 and 8 weeks of age. Renal tissues from *Tsc2*^Δ*podocyte*^ and control mice at 4, 6, and 8 weeks of age were stained with periodic acid-Schiff (PAS). Representative glomeruli from *Tsc2*^Δ*podocyte*^ and control mice are shown. Various degrees of glomerulosclerosis, partial glomerulosclerosis and protein casts in tubules (*asterisks*), and glomerulosclerosis with synechia formation (*arrowhead*) are shown. Scale bar: 50 μm. (**B**) Transmission electron microscopy (TEM) shows partial flattening and disorganization of podocyte foot processes (*arrows*). Scale bar: 5 μm. (**B, bottom**) Glomerular basement membrane thickness in *Tsc2*^Δ*podocyte*^ at 4 weeks of age was comparable with that in age-matched control mice (within *arrowheads*). Scale bar: 1 μm.

The kidney weight of *Tsc2*^Δ*podocyte*^ mice was comparable with that of control mice at 4 weeks of age (*Tsc2*^Δ*podocyte*^: 0.38 ± 0.08 g, *n* = 16; *Tsc2*^*flox/flox*^: 0.33 ± 0.12 g, *n* = 30; *P* = 0.87), whereas significant differences were observed at 8 weeks of age (*Tsc2*^Δ*podocyte*^: 0.44 ± 0.10 g, *n* = 5; *Tsc2*^*flox/flox*^: 0.64 ± 0.08 g, *n* = 9; *P* < 0.01). Morphologically, podocytes of *Tsc2*^Δ*podocyte*^ mice underwent hypertrophy, and the podocyte foot process was lost at 4 weeks of age (Fig 3A and 3B). However, the thickness of the glomerular basement membrane was almost comparable in *Tsc2*^Δ*podocyte*^ mice and controls at 4 weeks of age (*Tsc2*^Δ*podocyte*^: 0.17 ± 0.05 μm; *Tsc2*^*flox/flox*^: 0.17 ± 0.04 μm; *P* = 0.98). The glomerulosclerosis index was higher in *Tsc2*^Δ*podocyte*^ mice than in control mice at 4 weeks of age and increased with age (S2A Fig). At 6 weeks of age, *Tsc2*^Δ*podocyte*^ mice showed an increased glomerular size, FSGS and proteinaceous casts (Fig 3A and S2B Fig). Interestingly, crescent formation was found in some glomeruli of *Tsc2*^Δ*podocyte*^ mice at 8 weeks of age; most of the crescents were fibrotic (Fig 3A and S2C Fig).

**Fig 4:**
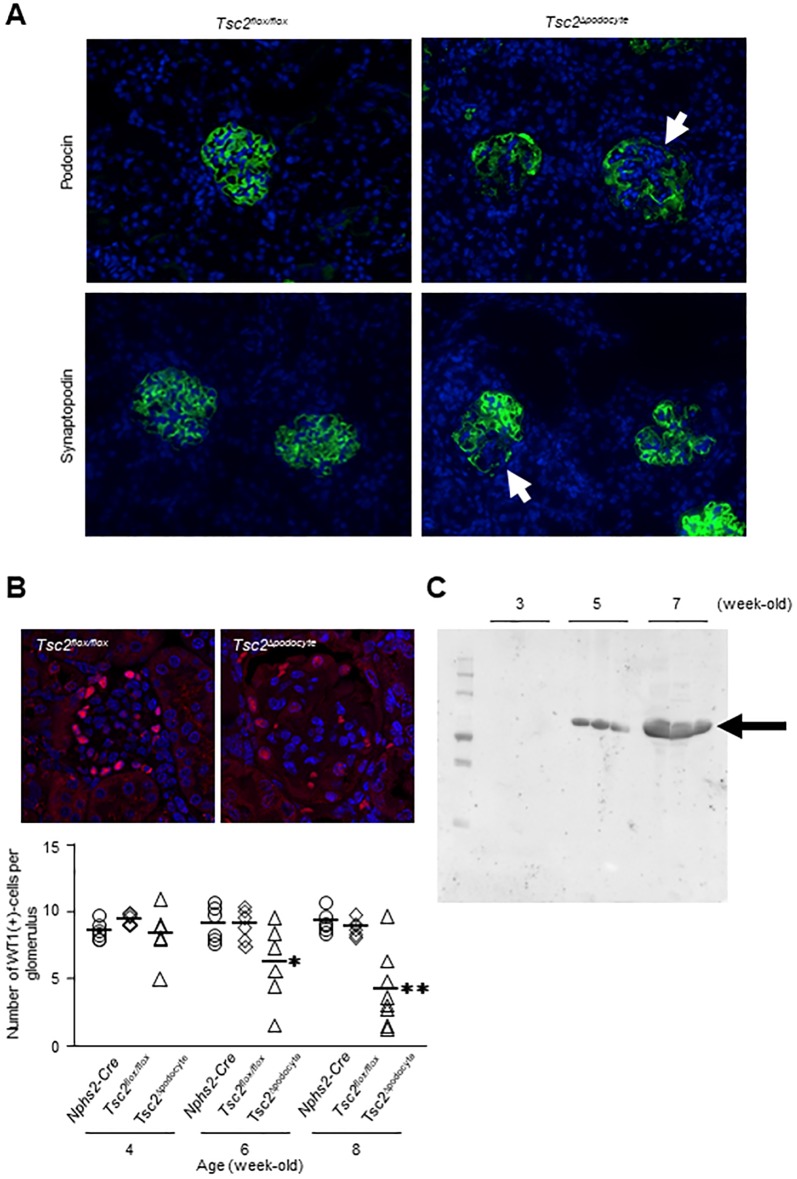
Podocytes in *Tsc2*^Δ*podocyte*^ mice were excreted in urine from glomeruli with progression of renal functional decline. (**A**) Immunofluorescence staining of podocin and synaptopodin showed decreased signal intensity in *Tsc2*^Δ*podocyte*^ mice (*arrows*). (**B, top**) The number of podocytes was decreased in the glomeruli of *Tsc2*^Δ*podocyte*^ mice. Representative images of WT1 positive (red) and Hoechst 33342-positive (blue) podocytes are shown. (**B, bottom**) The graph shows the average number of podocytes per glomerulus in each group. Each dot represents the mean number of WT1-positive cells in about 20 glomeruli from the indicated mouse, and horizontal lines represent the mean number of WT1-positive cells in each group. *Tsc2*^Δ*podocyte*^ mice had fewer WT1-positive podocytes per glomerulus compared with age-matched controls. *P < 0.05, **P < 0.01 compared with age-matched controls. (**C**) Western blot analysis of abundance of WT1 in urine from *Tsc2*^Δ*podocyte*^ mice. Loaded samples contained equal amounts of creatinine. WT1 gives a band at 52 kDa (*arrow*).

We next examined podocyte distribution in glomeruli using the podocyte markers podocin and synaptopodin. At 4 weeks of age, there were no obvious differences in the expression patterns of podocin and synaptopodin in the glomeruli of *Tsc2*^Δ*podocyte*^ and control mice. However, their expressions were lost in some glomeruli of *Tsc2*^Δ*podocyte*^ mice at 6 weeks of age (Fig 4A). We then determined the average number of podocytes per glomerulus by counting WT1-positive podocytes in *Tsc2*^Δ*podocyte*^ mice from 4 to 8 weeks of age. The number of podocytes in *Tsc2*^Δ*podocyte*^ mice at 6 to 8 weeks of age, but not at 4 weeks of age, was significantly decreased compared with control mice (Fig 4B). We further assessed the correlation between the number of WT1-positive podocytes and biochemical parameters and found that the number of WT1-positive podocytes in *Tsc2*^Δ*podocyte*^ mice was negatively correlated with the urinary ACR, BUN, TC and HDL-C and positively associated with serum albumin (S3 Fig). Furthermore, WT1-positive podocytes began to be excreted in the urine of *Tsc2*^Δ*podocyte*^ mice at 5 weeks of age (Fig 4C).

### *Tsc2* deficiency reduces autophagic activity in podocytes

**Fig 5:**
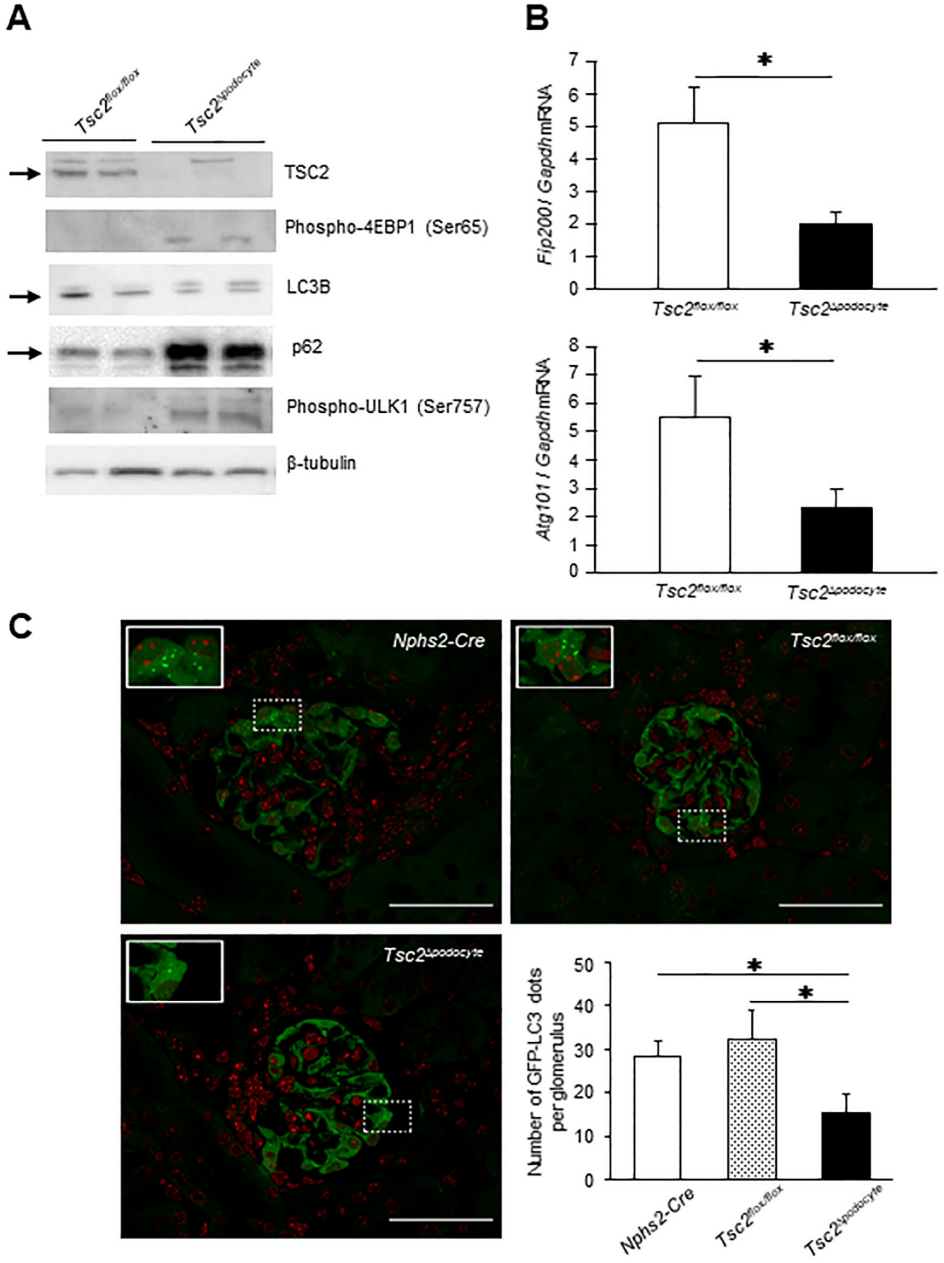
mTORC1 hyperactivation led to a decreased autophagic activity in the podocytes. (**A**) Primary cultured podocytes were isolated from *Tsc2*^Δ*podocyte*^ and wild-type controls, followed by western blot analyses of TSC2, phospho-4EBP1, LC3B type II, p62, phospho-ULK1, and β-tubulin. *Arrows* indicate specific bands corresponding to each indicated protein. (**B**) *FIP200* and *ATG101* mRNA expressions were decreased in the primary cultured podocytes from *Tsc2*^Δ*podocyte*^. (**C**) Representative fluorescence images of glomeruli of *Nphs2-Cre*- (**top, left**), *Tsc2*^*flox/flox*^- (**top, right**) and *Tsc2*^Δ*podocyte*^-GFP-LC3 transgenic mice (**bottom, left**) at 4 weeks of age. The white box indicates the location of the magnified figure. Scale bar: 50 μm. (**bottom, right**) Quantitative analysis of autophagic activity *in vivo*. Graph bars show the number of GFP-LC3 dots per glomerulus from the indicated mice (*n* = 3 per group). The number of GFP-LC3 dots per glomerulus was counted in 20 independent visual fields from the indicated mice. Results are expressed as means ± s.d. *P < 0.05 compared with age-matched controls.

To explore the molecular mechanism of podocyte dysfunction in *Tsc2*^Δ*podocyte*^ mice, we conducted microarray analysis using the total RNA of primary podocytes isolated from *Tsc2*^Δ*podocyte*^ and control *Tsc2*^*flox/flox*^ mice. We found that 858 genes were differentially expressed between these groups (fold-change difference ≥ |1.5|, *P* < 0.05, S4A Fig). IPA analysis of the differentially expressed genes showed significant enrichment for pathways involved in glycolysis I, gluconeogenesis I, NRF2 (nuclear factor, erythroid derived 2, like 2)-mediated oxidative stress response, glutathione-mediated detoxification, SPINK1 general cancer pathway, and MIF regulation of innate immunity (S4B Fig). In addition, the network analysis in IPA mapped the significant genes to network in mTOR signalling activating pathway, in which an inhibition of autophagy regulation is predicted (S4C Fig). Taken together, we hypothesized that *Nrf2* may be activated in the podocytes of *Tsc2*^Δ*podocyte*^ mice. NRF2 is a transcription factor that translocates to the nucleus in response to oxidative stress to activate the transcription of various detoxifying enzymes [24]. Moreover, the *Nrf2/Keap1* ubiquitination and degradation system is associated with the phosphorylation of p62, which is an autophagy-related molecule that is also modulated by mTORC1 activity [25]. Accordingly, we speculate that mTORC1 inhibits autophagic degradation and increase the intracellular level of p62, leading to noncanonical activation of *Nrf2* in the podocytes of *Tsc2*^Δ*podocyte*^ mice. The level of p62 was substantially increased in the podocytes of *Tsc2*^Δ*podocyte*^ mice compared with control mice (Fig 5A). Decreased formation of LC3 type II, an autophagy-related protein, was also observed in the podocytes of *Tsc2*^Δ*podocyte*^ mice, concomitant with the increased phosphorylation of ULK1 at Ser757 and 4EBP1 at Ser65 (Fig 5A). In addition, *FIP200* and *ATG101* genes, which are involved in the initiation of autophagy, were significantly decreased in the podocytes of *Tsc2*^Δ*podocyte*^ mice compared with control mice (Fig 5B). Finally, we crossed *Tsc2*^Δ*podocyte*^ mice with GFP-LC3 transgenic mice (GFP-LC3 Tg) to evaluate autophagic activity *in vivo*. The number of GFP-LC3 puncta was significantly decreased in *Tsc2*^Δ*podocyte*^ mice at 4 weeks of age compared with age-matched control mice (Fig 5C).

### Rapamycin treatment extends survival in *Tsc2*^Δ*podocyte*^ mice

**Fig 6:**
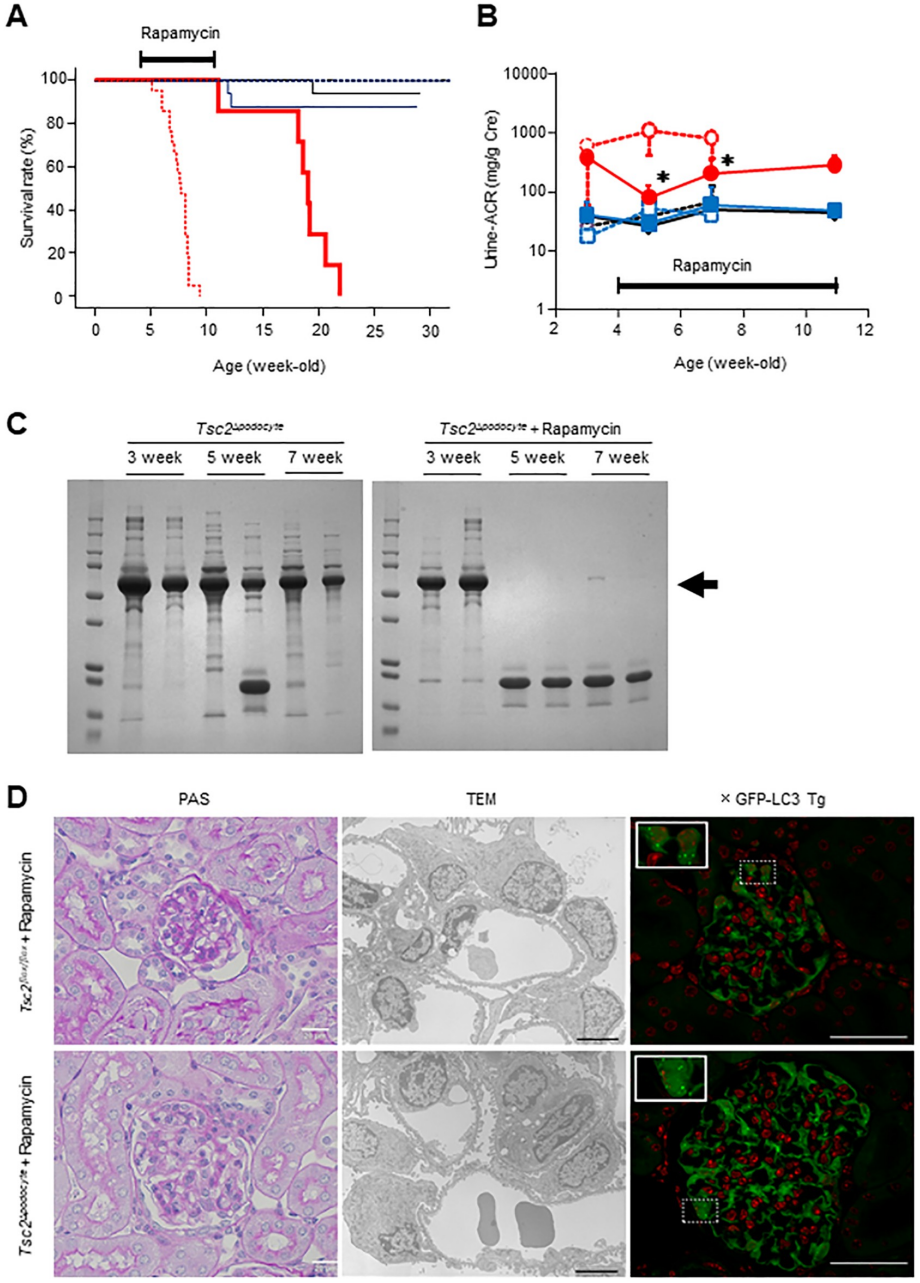
Rapamycin prevented death from renal dysfunction in *Tsc2*^Δ*podocyte*^. (**A**) *Tsc2*^Δ*podocyte*^ mice treated with rapamycin had improved survival compared to vehicle-treated *Tsc2*^Δ*podocyte*^ mice. From 4 to 11 weeks of age, rapamycin was injected intraperitoneally (2 mg/kg body weight) twice a day (*bold vertical line*). *Nphs2-Cre* with rapamycin treatment (*black line*), *n* = 17; *Tsc2*^*flox/flox*^ with rapamycin treatment (*blue line*), *n* = 16; *Tsc2*^Δ*podocyte*^ with rapamycin treatment (*red line*), *n* = 8; vehicle-treated *Nphs2-Cre* (*dashed black line*), *n* = 7; vehicle-treated *Tsc2*^*flox/flox*^ (*dashed blue line*), *n* = 9; vehicle-treated *Tsc2*^Δ*podocyte*^ (*dashed red line*), *n* = 26. (**B**) Eight-week follow-up of rapamycin-treated *Tsc2*^Δ*podocyte*^ for proteinuria (*n* = 6 per group). *P < 0.05 compared with age-matched vehicle-treated *Tsc2*^Δ*podocyte*^. (**C**) Urine collected from vehicle-treated and rapamycin-treated *Tsc2*^Δ*podocyte*^ at 3, 5, and 7 weeks of age was subjected to SDS-PAGE. *Arrow* indicates the bands corresponding to albumin. (**D, left**) Renal tissues from *Tsc2*^Δ*podocyte*^ and control mice with rapamycin treatment for 7 weeks were stained with PAS. Rapamycin treatment decreased the large amounts of PAS-positive materials present in the mesangial area of *Tsc2*^Δ*podocyte*^. Scale bar: 20 μm. (**D, center**) TEM analysis of rapamycin-treated *Tsc2*^*flox/flox*^ and *Tsc2*^Δ*podocyte*^. Rapamycin treatment restored podocyte hypertrophy and foot process effacement in *Tsc2*^Δ*podocyte*^ at 11 weeks of age. Scale bar: 4 μm. (**D, right**) Representative fluorescence images of glomerulus in *Tsc2*^*flox/flox*^ and *Tsc2*^Δ*podocyte*^ mice mated with GFP-LC3 transgenic mice 1 week after rapamycin treatment are shown. The white box indicates the location of the magnified figure. Scale bar: 50 μm.

Next, we evaluated the effects of rapamycin in *Tsc2*^Δ*podocyte*^ mice. Rapamycin, an inhibitor of mTORC1, was administered via intraperitoneal injection. Rapamycin impaired proteinuria and extended the survival of *Tsc2*^Δ*podocyte*^ mice, although *Tsc2*^Δ*podocyte*^ mice exhibited renal dysfunction and died by 10 weeks after birth without rapamycin treatment, as revealed above (Fig 6A and 6B). Albuminuria vanished 1 week after rapamycin treatment (Fig 6C). *Tsc2*^Δ*podocyte*^ mice exhibited significantly higher levels of BUN and SCr. However, rapamycin treatment decreased these levels to those comparable to the control (S3 Table). Morphologically, rapamycin treatment restored podocyte hypertrophy, foot process effacement, FSGS and proteinaceous casts (Fig 6D). We next conducted microarray analysis using the total RNA of primary podocytes isolated from rapamycin-treated *Tsc2*^Δ*podocyte*^ mice. We found expression of 858 genes were significantly differed in *Tsc2*^Δ*podocyte*^ mice compared with control, and also found that expression of 810 genes out of the 858 genes was normalized in rapamycin-treated *Tsc2*^Δ*podocyte*^ mice (S5A Fig). Furthermore, the levels of intracellular p62 and LC3 type II in podocytes isolated from rapamycin-treated *Tsc2*^Δ*podocyte*^ mice were comparable to those from control mice (S5B Fig). There were no apparent differences in the number of GFP-LC3 puncta between these groups, implying that *in vivo* autophagic activity was also restored in the podocytes of rapamycin-treated *Tsc2*^Δ*podocyte*^ mice (Fig 6D and S5C Fig).

### mTORC1 is activated in podocytes in patients with CKD

**Fig 7:**
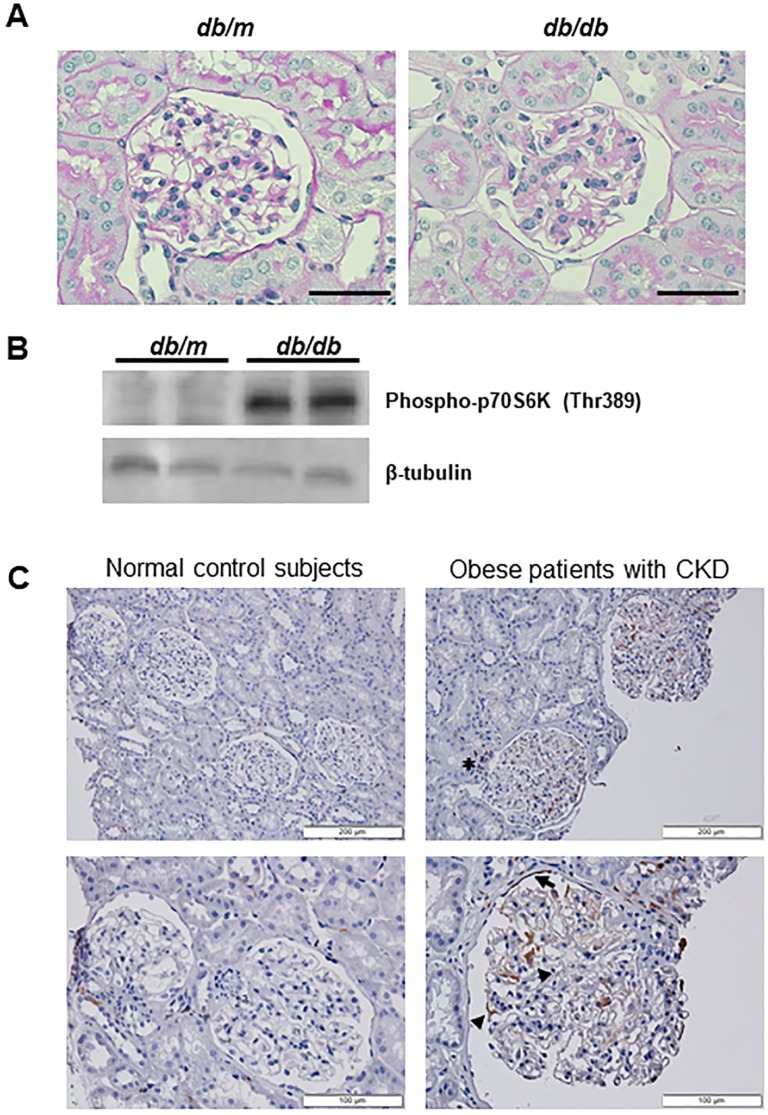
mTORC1 is activated in podocytes in patients with CKD. (**A**) Representative photomicrographs of periodic acid-Schiff (PAS) staining in the kidney of *db/db* and *db/m* mice. *Db/db* mice exhibited glomerulosclerosis at 24 weeks of age. Scale bar: 50 μm. (**B**) Western blot analysis of p70 S6 kinase (p70S6K) phosphorylation using primary cultured podocytes isolated from *db/db* and *db/m* mice at 24 weeks of age. β-tubulin served as the internal control. (**C**) mTORC1 is activated in glomeruli of obese patients with CKD. Human kidney biopsy specimens from normal controls and obese patients with CKD were immunostained with anti-phospho-S6 ribosomal protein (pS6) antibody and counterstained with hematoxylin. pS6 protein was detected in podocytes (*arrowheads*), parietal cells lining Bowman’s capsule (*arrow*), and tubulointerstitial regions (*asterisk*) of obese patients with CKD. Representative low-magnification (**top**) and high-magnification (**bottom**) images are shown.

To explore whether mTORC1 activity is associated with obesity-related renal functional decline, we examined mTORC1 activation in *vivo*. First, we examined mTORC1 activation in kidney of *db/db* mice, used as an obese model of genetic diabetes, at 24 weeks of age. The *db/db* mice showed a significantly higher urinary ACR compared with age-matched *db/m* mice (*db/db*: 574 ± 175 mg g^-1^ creatinine; *db/m*: 22 ± 3 mg g^-1^ creatinine; *n* = 5/group; *P* < 0.01) and featured glomerulosclerosis at 24 weeks of age (Fig 7A). We found that phosphorylation of p70 S6 kinase, a direct phosphorylation target of mTORC1, was enhanced in primary cultured podocytes isolated from *db/db* mice (Fig 7B). We further examined mTORC1 activation in renal biopsy specimens from obese patients with CKD. The obese patients with CKD were normoglycemic, similar to normal controls (S4 Table). Biopsy specimens from obese patients with CKD showed glomerulomegaly (approximate glomerular size > 250 μm) (Fig 7C). pS6 protein was detected in podocytes, the parietal cells lining Bowman’s capsule and tubulointerstitial regions of obese patients with CKD (Fig 7C). The ratio of pS6-positive glomeruli was significantly higher in the glomeruli of obese patients with CKD than in control individuals (obese patients with CKD: 81 ± 16%; normal controls: 27 ± 24%; *P* = 0.03).

## Discussion

This study revealed that podocyte-specific deletion of *Tsc2* contributes to severe podocyte injury, leading to massive proteinuria, end-stage renal dysfunction and increased mortality. *Tsc2*^Δ*podocyte*^ mice were normoglycemic and nonobese but showed an increased glomerular size, glomerulosclerosis, proteinaceous casts, crescent formation and increased tubulointerstitial fibrotic lesions, with a pattern that was similar to that of FSGS in humans. Recent work reported that podocyte-specific *Tsc1* knockout mice, which lack the TSC1-TSC2 heterodimer complex, exhibit structural abnormalities such as FSGS with occasional crescent formation and podocyte vacuolation [26]. Additionally, podocyte-specific *Tsc1* knockout mice show features of diabetic nephropathy and mTORC1 hyperactivation is present in podocytes of patients with diabetic nephropathy [27, 28]. In addition to the above findings, we found an increase in the number of mitochondria in the podocytes of *Tsc2*^Δ*podocyte*^ mice. Similar findings were reported in cardiac-specific *Tsc2*-deficient mice, which showed structural abnormalities of mitochondria, although the mitochondrial function was maintained [29].

To explore the underlying mechanisms of the renal functional decline in *Tsc2*^Δ*podocyte*^ mice, we performed microarray analysis, finding that *Tsc2* deletion in podocytes may modulate the *Nrf2*-mediated oxidative stress response pathway (S4B Fig). We also revealed an increased abundance of p62 and a decreased abundance of LC3B type II in the podocytes of *Tsc2*^Δ*podocyte*^ mice by suppressing autophagic activity through the mTOR-ULK1 pathway (Fig 5). We further revealed that inhibition of mTORC1 activity in the podocytes of *Tsc2*^Δ*podocyte*^ mice by rapamycin injection attenuated the podocyte dysfunction, including the impaired autophagic activity and structural abnormalities, preventing the massive proteinuria, end-stage renal dysfunction and increased mortality seen in controls. Considering these findings, we conclude that mTORC1 hyperactivation in podocytes could impair the autophagy and cause cytoplasmic accumulation of p62, leading to *Nrf2* activation via dissociation of the *Nrf2/Keap1* complex [25]. Autophagy is a conserved mechanism of intracellular degradation that maintains homeostasis and cell integrity and its dysregulation has been suggested to cause a variety of disease processes [30]. Podocytes exhibit an unusually high level of constitutive autophagy, and a recent report showed that podocyte-specific deletion of the *Atg5* gene which is known as one of the autophagy conjugation systems led to podocyte injury such as proteinuria, foot process effacement, vacuolation and progressive development of glomerulosclerosis, which are similar to the structural abnormalities observed in *Tsc2*^Δ*podocyte*^ mice [31, 32]. However, podocyte-specific *Atg5*-deficient mice did not exhibit end-stage renal dysfunction and increased mortality, which is inconsistent with the characteristics of *Tsc2*^Δ*podocyte*^ mice. Zhou *et al*. [33] reported that mTORC1 exerts a dual inhibitory effect on autophagy, blocking autophagy not only at the initiation stage via suppression of the ULK1 complex, but also at the degradation stage via inhibition of lysosomal function. One possible explanation for the severe characteristics of *Tsc2*^Δ*podocyte*^ mice may be a dual suppressive effect of mTORC1 on autophagy, leading to severe podocytopathy. However, functional investigations are required.

Obesity leads to CKD. Moreover, obese patients show proteinuria and some patients have nephrotic-range proteinuria and progressive loss of renal function [34]. The pathologic features of obese patients with CKD include glomerulomegaly and FSGS [35, 36], and these features were observed in the renal biopsy specimens analysed in the present study (Fig 7C). To further investigate the involvement of mTORC1 in obesity-related kidney dysfunction, we observed mTORC1 activity in renal biopsy specimens from obese patients with CKD. As shown in Fig 7C, obese patients with FSGS exhibited an increase in mTORC1 activity in podocytes and the parietal cells of Bowman’s capsule, in contrast to nonobese patients. An increased mTORC1 activity has been reported in cellular crescents from patients with crescentic glomerular diseases [26]. An increased mTORC1 activity in podocytes and the parietal cells of Bowman’s capsule may be related to crescent and scar formation in CKD, but the underlying mechanisms remain to be resolved. We also found increased mTORC1 activity in tubulointerstitial regions of obese patients with CKD (Fig 7C). Recently, van der Heijden *et al*. [37] reported that high-fat diet-challenged mice exhibited upregulation of pro-inflammatory genes and infiltrating macrophages in the tubulointerstitium. High-fat diet-induced obesity may cause the infiltration of macrophages into tubulointerstitial regions accompanied by the activation of mTORC1, leading to chronic low-grade inflammation and renal functional decline. Nonetheless, further experimental investigations are required.

The major limitation of the current study is the lack of information on the pathogenesis of mTORC1 activation in podocytes from obese patients with CKD. mTORC1 is an important factor in protein synthesis that is activated by amino acids. Recent reports showed that increased levels of branched-chain amino acids (BCAA) were associated with T2DM and obesity [38]. Furthermore, Giesbertz *et al*. [39] reported increased levels of BCAA and α-ketoisocaproic acid, the transamination product of leucine, in plasma of *db/db* mice and that adipose tissues contribute most to the changes in plasma BCAA. Obese mice show a decreased protein level and activity of the mitochondrial BCAA transferase and the rate-limiting branched-chain keto acid dehydrogenase complex [40]. Therefore, disturbed expression of genes related to the metabolism of amino acids in adipose tissue may significantly contribute to the metabolism of BCAA, leading to the activation of mTORC1 in podocytes. Disturbed expression of cytokines and growth factors could be another causative factor for obesity-related kidney dysfunction. Inflammatory cytokines are modulated in the glomeruli of obesity-related glomerulopathy [41]. Lee *et al*. [42] also reported that IκB kinase β, a downstream kinase in the tumor necrosis factor α-signalling pathway, phosphorylates TSC1, resulting in the activation of mTORC1. However, it is uncertain whether the levels of tumor necrosis factor α or other cytokines were increased in the obese patients with CKD examined in this study because of the sample limitations. In addition, it is difficult to dissect out the individual contributions of obesity and T2DM to renal functional decline. Indeed, mTORC1 target genes and mTOR mRNA itself were reported to be induced in glomeruli from patients with diabetic nephropathy [28]. We further analysed the levels of *Tsc1* and *Tsc2* mRNA in diabetic nephropathy using the Nephroseq database (https://www.nephroseq.org) and found that *Tsc2* mRNA was also significantly decreased in both glomeruli (Glom) and the tubulointerstitium (TubInt) from patients with diabetic nephropathy (Glom in healthy living donor: 0.92 ± 0.31; Glom in diabetic nephropathy: 0.71 ± 0.46 [*P* = 0.002]; TubInt in healthy living donor: 0.13 ± 0.24; TubInt in diabetic nephropathy: -0.02 ± 0.27 [*P* = 0.02]). However, in this study, we revealed *Tsc2*^Δ*podocyte*^ mice were normoglycemic and nonobese but showed a similar histological pattern of FSGS in obese patients with CKD, which has not been reported in the analyses of podocyte-specific *Tsc1* knockout mice [26, 27]. Moreover, we have also found that mTORC1 is activated in podocytes of nondiabetic obese patients with CKD, so an evaluation of the involvement of the *Tsc2* gene in nondiabetic obese patients with CKD might provide valuable clues for understanding the pathogenesis of obesity-related renal diseases.

In conclusion, mTORC1 hyperactivation in podocytes leads to severe renal dysfunction caused by the induction of oxidative stress and impairment of autophagic activity in podocytes. mTORC1 may play important roles in maintaining podocyte functions, and inhibition of mTORC1 activity in podocytes could be a key therapy for obesity-related kidney dysfunction.

## Supporting information

S1 Fig Analysis of podocyte-specific *Tsc2* knockout mice (*Tsc2*^Δ*podocyte*^) in C57BL/6 strain. (**A-D**) Twenty-four-week follow-up of *Tsc2*^Δ*podocyte*^ (*solid line*) and homozygous floxed *Tsc2* mice (*Tsc2*^*flox/flox*^, *dashed line*) for urine albumin-to creatinine ratio (**A**), serum albumin (**B**), total cholesterol (**C**) and HDL-cholesterol (**D**) (*n* = 3-5/each group). ACR, albumin-to creatinine ratio; ALB, albumin; TC, total cholesterol; HDL-c, HDL-cholesterol; dotted bars, *Tsc2*^*flox/flox*^; black bars, *Tsc2*^Δ*podocyte*^. (**E**) Kaplan-Meier survival plots for *Tsc2*^Δ*podocyte*^ (*solid line*) and control *Tsc2*^*flox/flox*^ mice (*dashed line*). The results were expressed as mean ± SD. *P < 0.05 to the age-matched control.
(PDF)

S2 Fig *Tsc2*^Δ*podocyte*^ showed an increased glomerular size and focal segmental glomerulosclerosis. (**A**) Morphometric analysis using PAS-stained renal tissues. Fifty glomeruli were randomly selected from the indicated mice (*n* = 3/group) and their glomerulosclerosis indices assessed. The bar graph shows the glomerulosclerosis index in *Nphs2-Cre* mice (*white bars*), *Tsc2*^*flox/flox*^ mice (*dotted bars*) and *Tsc2*^Δ*podocyte*^ mice (*black bars*). The results are expressed as the mean ± s.d. *P < 0.05 versus the age-matched control. (**B**) *Tsc2*^Δ*podocyte*^ mice show increased glomerular size. Renal sections from *Tsc2*^Δ*podocyte*^ and control mice at 6 weeks of age were stained with periodic acid-Schiff. Twenty glomeruli were randomly selected in each mouse (*n* = 3/genotype), and glomerular diameters were measured by using ImageJ. The results are expressed as the mean ± s.d. *P < 0.05 versus the age-matched control. (**C**) Renal sections from *Tsc2*^Δ*podocyte*^ and control mice at 8 weeks of age were stained with Masson’s trichrome. Scale bar: 100 μm.
(PDF)

S3 Fig Correlation between biochemical parameters and the numbers of WT1-positive podocytes per glomerulus. The numbers of podocytes were counted in each glomerulus of 3, 5 and 7 weeks of age *Tsc2*^Δ*podocyte*^ and control mice. The X-axis shows (**A**) the urine albumin-to-creatinine ratio (ACR), (**B**) blood urea nitrogen (BUN), (**C**) total cholesterol (TC), (**D**) HDL-cholesterol (HDL-c) and (**E**) serum albumin (ALB). △, *Nphs2-Cre*; □, *Tsc2*^*flox/flox*^; ○, *Tsc2*^Δ*podocyte*^ (*n* = 6/group).
(PDF)

S4 Fig Microarray analysis of primary podocytes isolated from *Tsc2*^Δ*podocyte*^ and control *Tsc2*^*flox/flox*^ mice. (**A**) Volcano plot showing top differentially expressed genes among *Tsc2*^Δ*podocyte*^ and *Tsc2*^*flox/flox*^ mice. (**B**) The significantly expressed genes between *Tsc2*^Δ*podocyte*^ and *Tsc2*^*flox/flox*^ mice were inputted to IPA for pathway enrichment analysis. Of these genes, IPA analysis further identified 625 genes, 388 of which were significantly increased and 237 of which were significantly decreased in *Tsc2*^Δ*podocyte*^ mice. The figure shows some of the top pathways identified by IPA (–log[*P*-value], >1.3; *z*-score, >2.0; threshold value, 0.05). *P*-values here are from right-tailed Fisher’s exact test. (**C**) Network analysis on differentially expressed genes between *Tsc2*^Δ*podocyte*^ and *Tsc2*^*flox/flox*^ mice mapped to networks involved in the mTOR signaling activating pathway.
(PDF)

S5 Fig Differential expression analysis in podocytes isolated from *Tsc2*^Δ*podocyte*^ and rapamycin-treated *Tsc2*^Δ*podocyte*^ mice (Rapa-*Tsc2*^Δ*podocyte*^). (**A**) Expression of 858 genes were significantly different in *Tsc2*^Δ*podocyte*^, and 810 out of 858 genes were normalized in rapamycin-treated *Tsc2*^Δ*podocyte*^ mice. Rapamaycin-treatment also caused disturbed expression of 76 genes in Rapa- *Tsc2*^Δ*podocyte*^ mice, although those levels were similar both in *Tsc2*^Δ*podocyte*^ and *Tsc2*^*flox/flox*^ mice. (**B**) Primary cultured podocytes were isolated from *Tsc2*^Δ*podocyte*^ mice 1 week after rapamycin treatment, followed by western blot analyses of LC3B type II, p62 and phospho-ULK1 (Ser757). The *arrow* indicates the band corresponding to LC3B type II. β-tubulin served as the internal control. (**C**) The graph bars show the number of GFP-LC3 puncta in each glomerulus from *Tsc2*^*flox/flox*^- and *Tsc2*^Δ*podocyte*^-GFP-LC3 transgenic mice. The results are expressed as the mean ± s.d. N.S., not statistically significant.
(PDF)

S1 Raw images Uncropped original images of gels and blots presented in the figures of this study. (PDF)

S1 Table Overview of the number of mice used in survival analyses. There were no mice that were euthanized before reaching the experimental endpoint. The numbers of mice that died without humane intervention and euthanized after reaching the experimental endpoint were also summarized.
(PDF)

S2 Table Characteristics of *Nphs2-Cre*, *Tsc2*^*flox/flox*^ and *Tsc2*^Δ*podocyte*^ mice. Data are expressed as mean ± SD (*n* = 10). Analysis of variance was used between groups; and multiple testing corrections were performed using the Tukey’s method. ACR, urine albumin to creatinin; BUN, blood urea nitrogen; Cre, creatinine; TP, total protein; ALB, alubumin; TC, total cholesterol; TG, triglyceride; HDL-c, high density lipoprotein-cholesterol. ^a^*P* < 0.05 vs. *Nphs2-Cre*, ^b^*P* < 0.05 *vs*. *Tsc2*^*flox/flox*^.
(PDF)

S3 Table Characteristics of rapamycin-treated *Nphs2-Cre*, *Tsc2*^*flox/flox*^ and *Tsc2*^Δ*podocyte*^ mice. Data are expressed as mean ± SD (*n* = 5). Analysis of variance was used between groups; and multiple testing corrections were performed using the Tukey’s method. There were no significant differences in the biochemical parameters among rapamycin-treated *Nphs2-Cre*, *Tsc2*^*flox/flox*^ and *Tsc2*^Δ*podocyte*^ mice. Abbreviations are as in S2 Table.
(PDF)

S4 Table Clinical characteristics of normal control subjects and patients diagnosed with FSGS perihilar variant. (PDF)

S1 Checklist The ARRIVE guidelines checklist. (DOCX)

## Abbreviations

4EBP1: eukaryotic translation initiation factor 4E- binding protein 1
ACR: albumin-to-creatinine ratio
BCAA: branched-chain amino acids
BUN: blood urea nitrogen
CKD: chronic kidney disease
FSGS: focal segmental glomerulosclerosis
HDL-C: HDL-cholesterol
mTOR: mammalian target of rapamycin
mTORC1: mammalian target of rapamycin complex 1
NRF2: nuclear factor, erythroid derived 2, like 2
PAS: periodic acid-Schiff
pS6: phospho-S6 ribosomal protein
SCr: serum creatinine
T2DM: type 2 diabetes mellitus
TC: total cholesterol
TSC1: tuberous sclerosis complex 1
TSC2: tuberous sclerosis complex 2
ULK1: unc-51-like kinase 1
WT1: Wilms tumor 1
