# Supplementary figures and images for: Podocyte-specific deletion of tubular sclerosis complex 2 promotes focal segmental glomerulosclerosis and progressive renal failure
Source: PLoS One. 2020 Mar 19;15(3):e0229397. doi: 10.1371/journal.pone.0229397 (PMC7082048; doi:10.1371/journal.pone.0229397)

S1 Fig. Iwata et al.

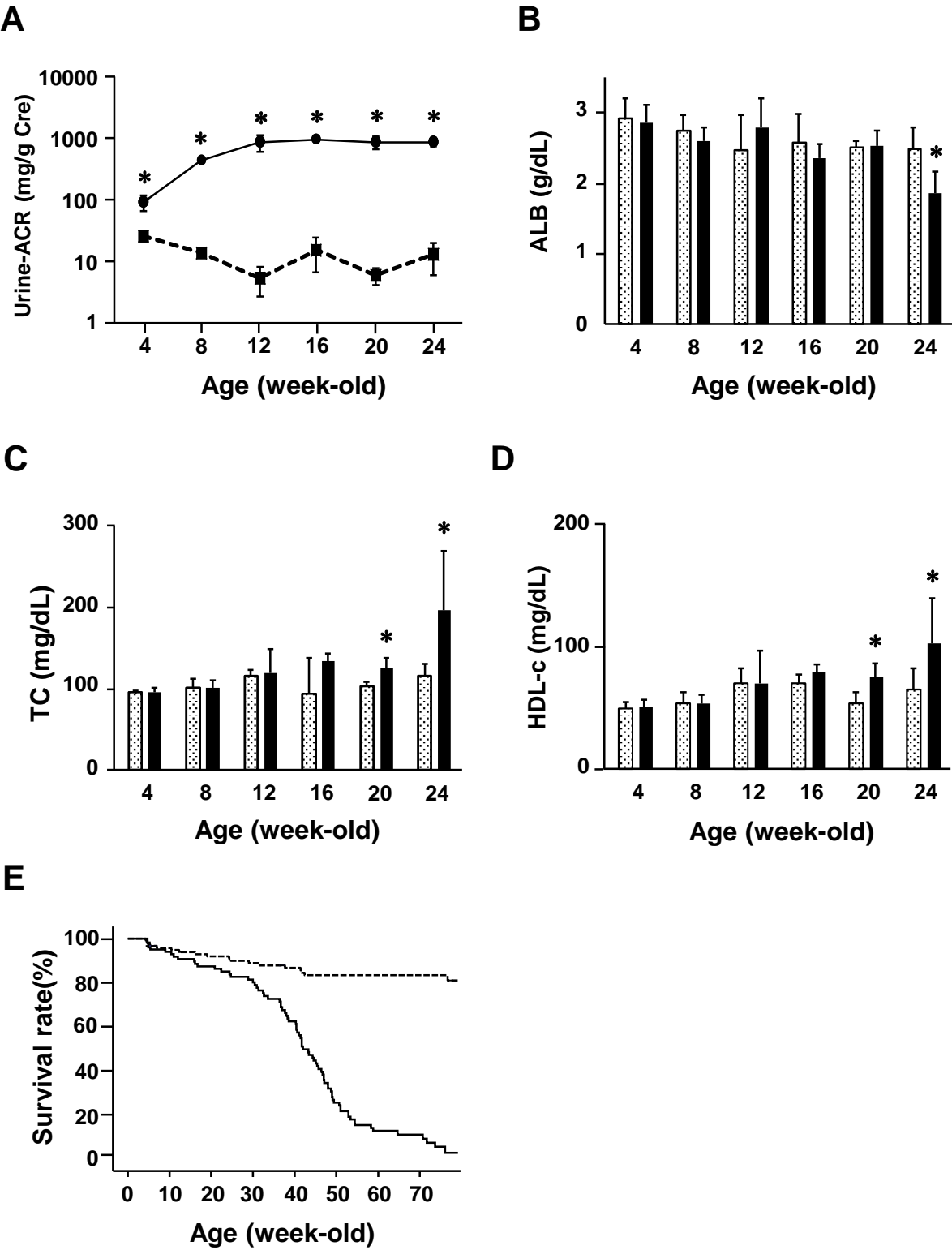

Supplement: S1 Fig — (A-D) Twenty-four-week follow-up of Tsc2Δpodocyte (solid line) and homozygous floxed Tsc2 mice (Tsc2flox/flox, dashed line) for urine albumin-to creatinine ratio (A), serum albumin (B), total cholesterol (C) and HDL-cholesterol (D) (n = 3-5/each group). ACR, albumin-to creatinine ratio; ALB, albumin; TC, total cholesterol; HDL-c, HDL-cholesterol; dotted bars, Tsc2flox/flox; black bars, Tsc2Δpodocyte. (E) Kaplan-Meier survival plots for Tsc2Δpodocyte (solid line) and control Tsc2flox/flox mice (dashed line). The results were expressed as mean ± SD. *P < 0.05 to the age-matched control. (PDF) [file pone.0229397.s001.pdf]

S2 Fig. Iwata et al.

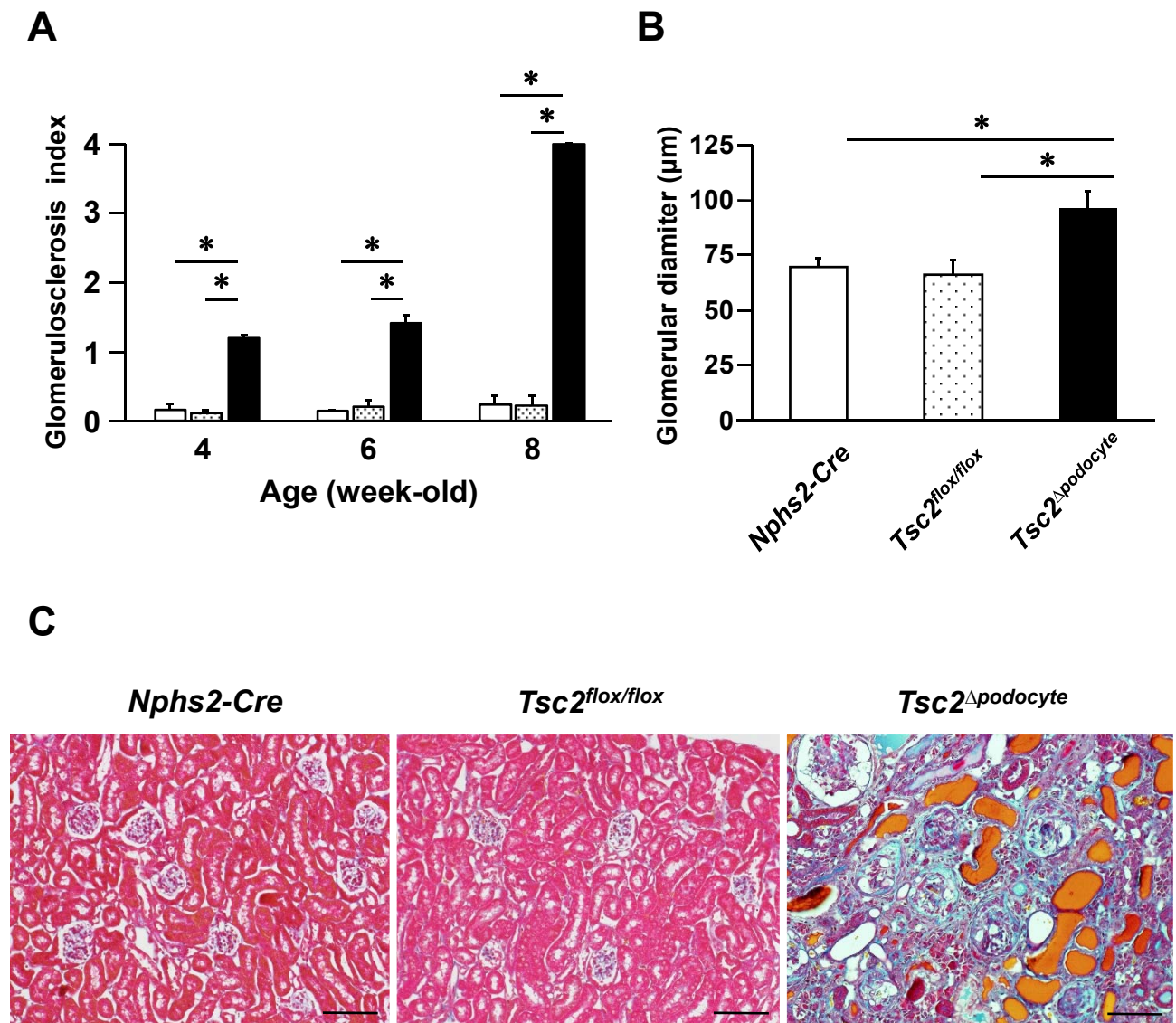

Supplement: S2 Fig — (A) Morphometric analysis using PAS-stained renal tissues. Fifty glomeruli were randomly selected from the indicated mice (n = 3/group) and their glomerulosclerosis indices assessed. The bar graph shows the glomerulosclerosis index in Nphs2-Cre mice (white bars), Tsc2flox/flox mice (dotted bars) and Tsc2Δpodocyte mice (black bars). The results are expressed as the mean ± s.d. *P < 0.05 versus the age-matched control. (B) Tsc2Δpodocyte mice show increased glomerular size. Renal sections from Tsc2Δpodocyte and control mice at 6 weeks of age were stained with periodic acid-Schiff. Twenty glomeruli were randomly selected in each mouse (n = 3/genotype), and glomerular diameters were measured by using ImageJ. The results are expressed as the mean ± s.d. *P < 0.05 versus the age-matched control. (C) Renal sections from Tsc2Δpodocyte and control mice at 8 weeks of age were stained with Masson’s trichrome. Scale bar: 100 μm. (PDF) [file pone.0229397.s002.pdf]

S3 Fig. Iwata et al.

**A**

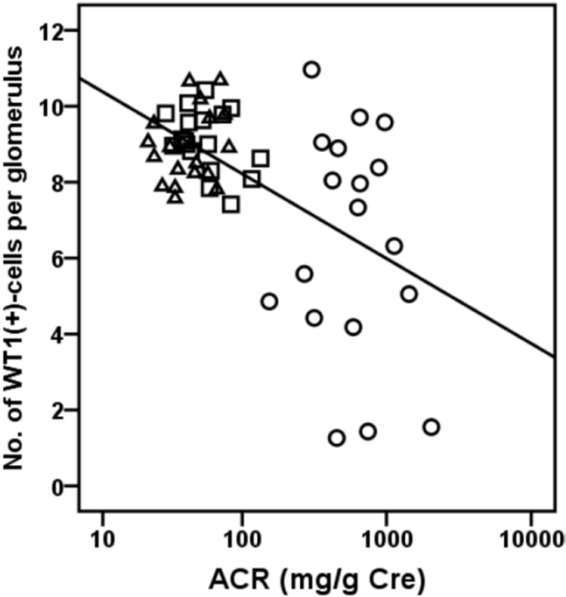

**B**

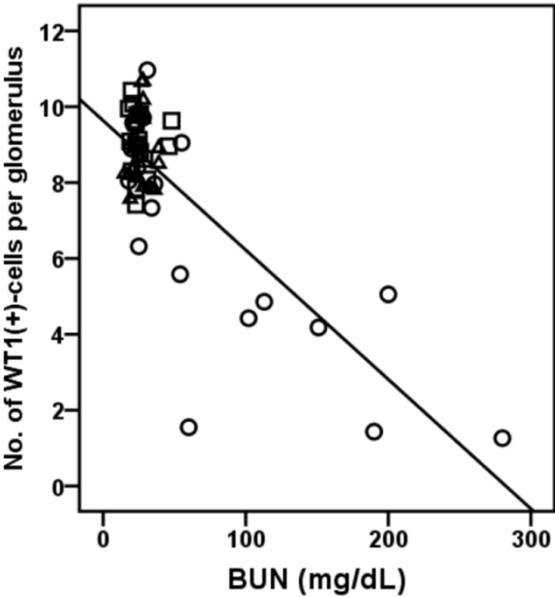

**C**

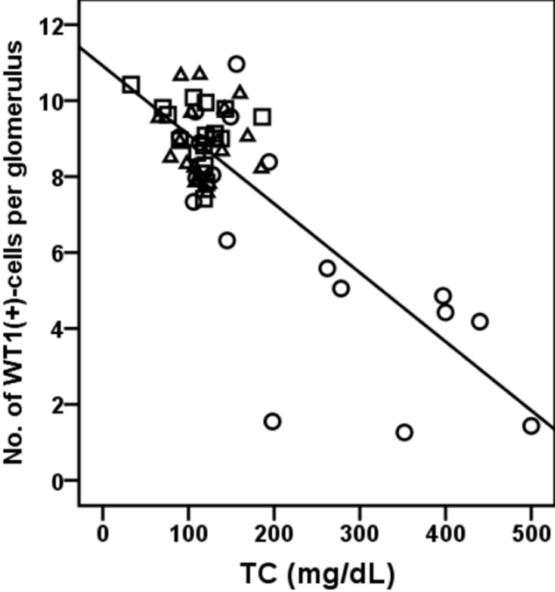

**D**

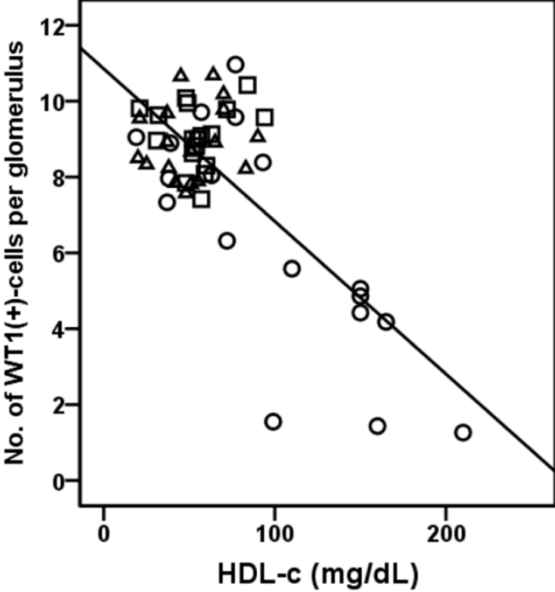

**E**

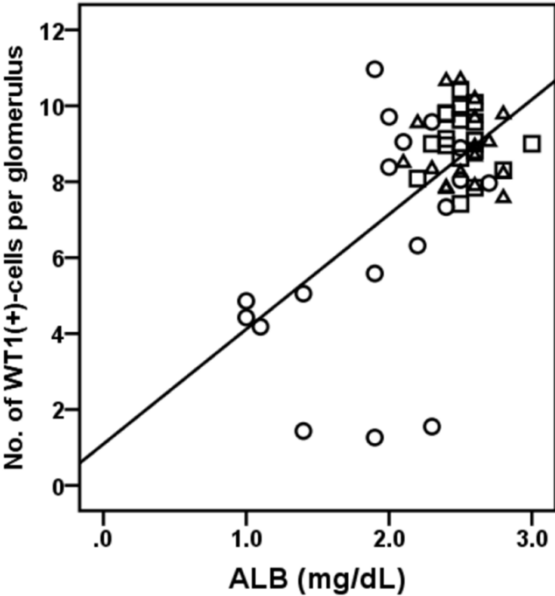

Supplement: S3 Fig — The numbers of podocytes were counted in each glomerulus of 3, 5 and 7 weeks of age Tsc2Δpodocyte and control mice. The X-axis shows (A) the urine albumin-to-creatinine ratio (ACR), (B) blood urea nitrogen (BUN), (C) total cholesterol (TC), (D) HDL-cholesterol (HDL-c) and (E) serum albumin (ALB). △, Nphs2-Cre; □, Tsc2flox/flox; ○, Tsc2Δpodocyte (n = 6/group). (PDF) [file pone.0229397.s003.pdf]

S4 Fig. Iwata et al.

A

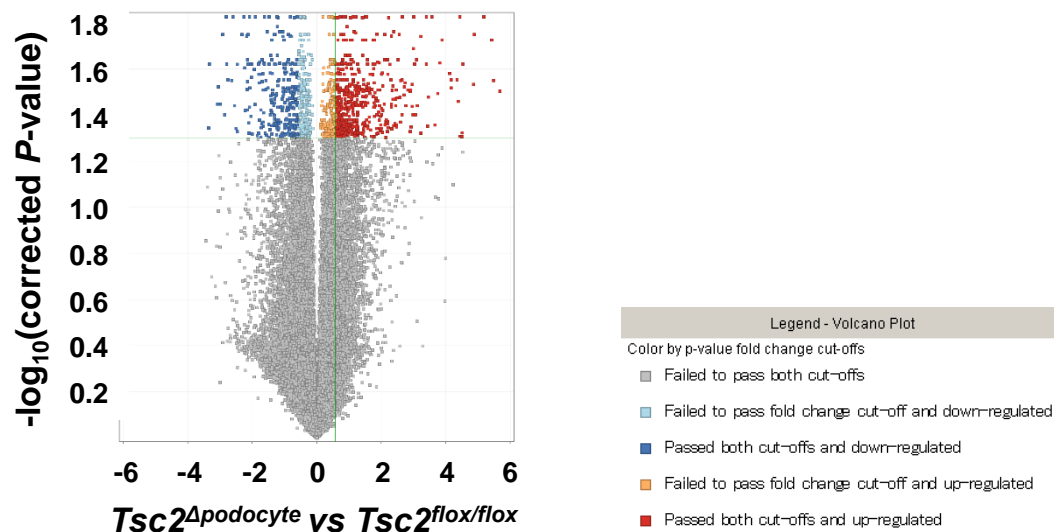

B

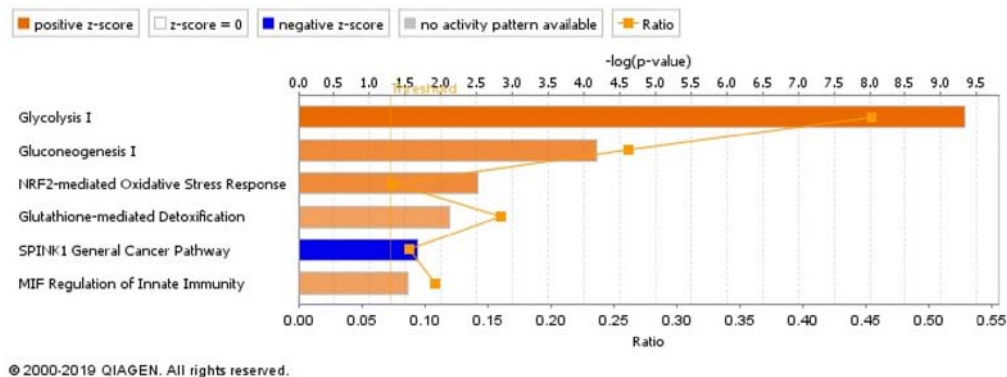

C

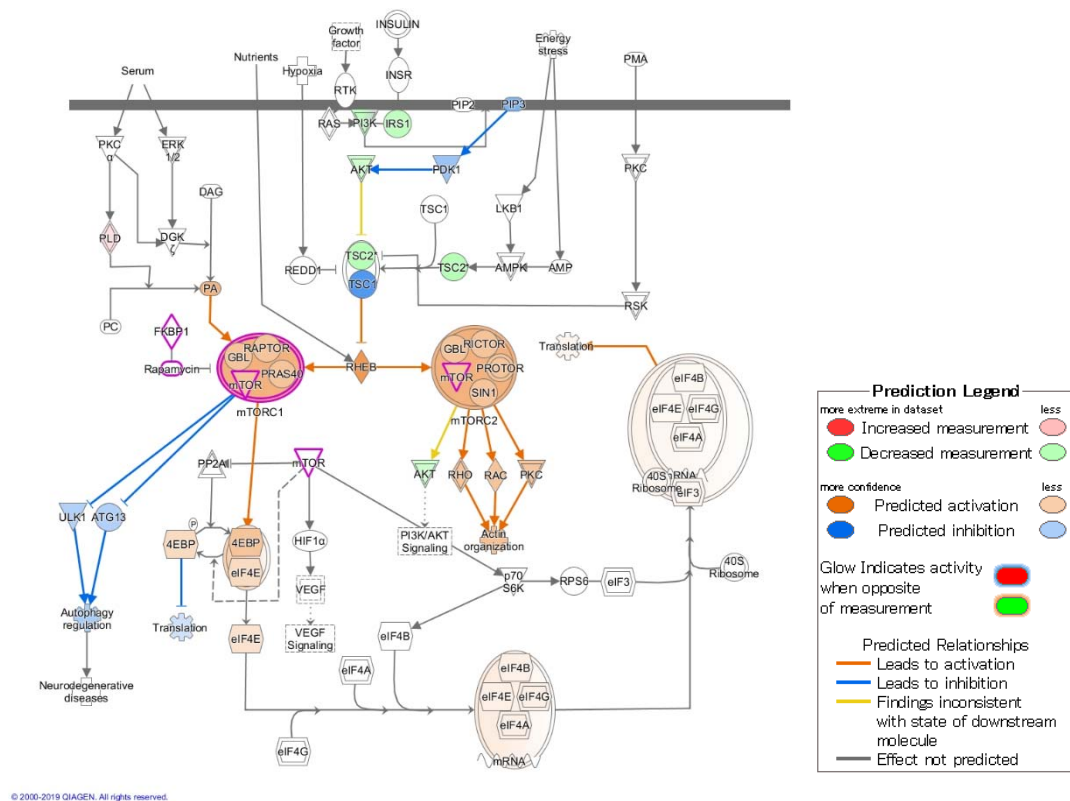

Supplement: S4 Fig — (A) Volcano plot showing top differentially expressed genes among Tsc2Δpodocyte and Tsc2flox/flox mice. (B) The significantly expressed genes between Tsc2Δpodocyte and Tsc2flox/flox mice were inputted to IPA for pathway enrichment analysis. Of these genes, IPA analysis further identified 625 genes, 388 of which were significantly increased and 237 of which were significantly decreased in Tsc2Δpodocyte mice. The figure shows some of the top pathways identified by IPA (–log[P-value], >1.3; z-score, >2.0; threshold value, 0.05). P-values here are from right-tailed Fisher’s exact test. (C) Network analysis on differentially expressed genes between Tsc2Δpodocyte and Tsc2flox/flox mice mapped to networks involved in the mTOR signaling activating pathway. (PDF) [file pone.0229397.s004.pdf]

**A**

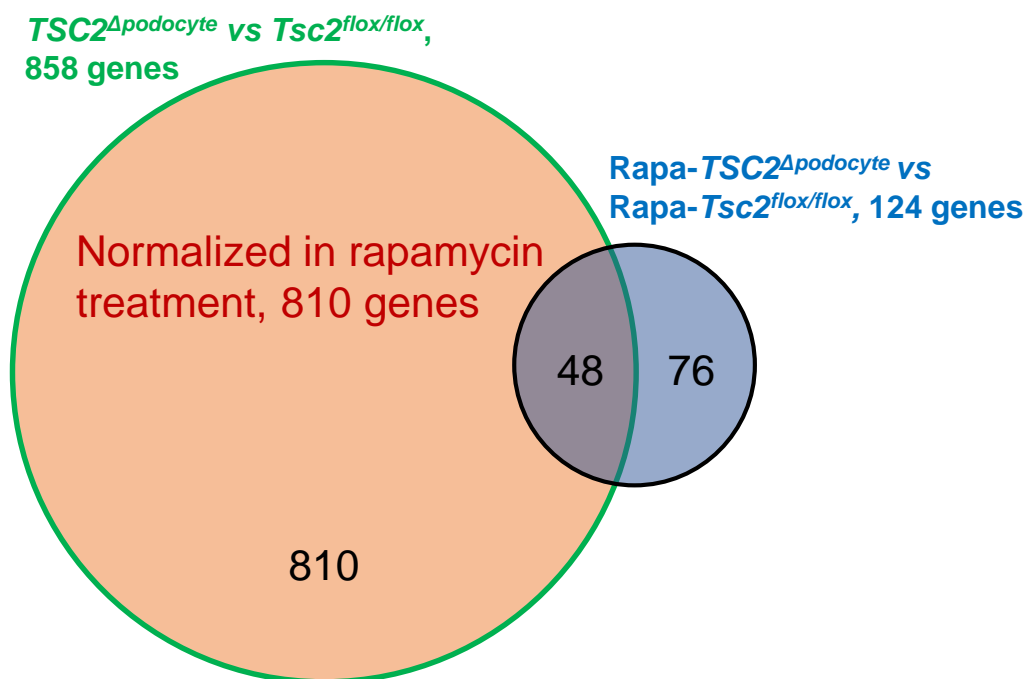

**B**

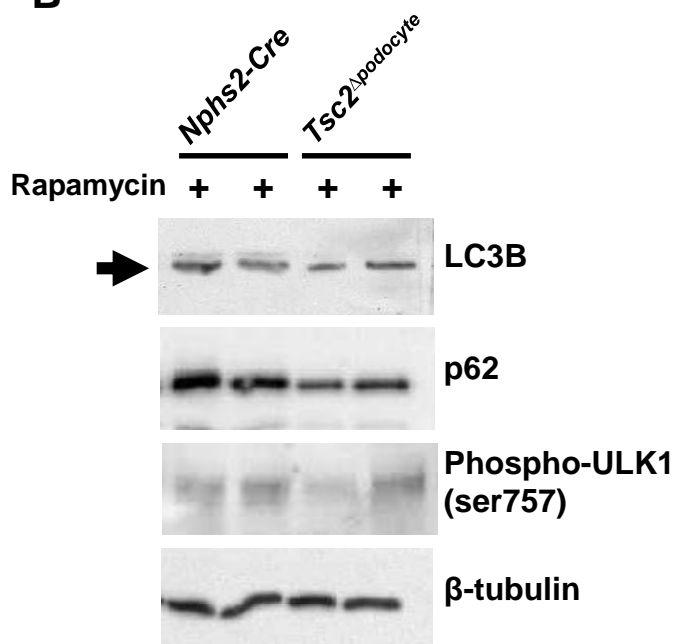

**C**

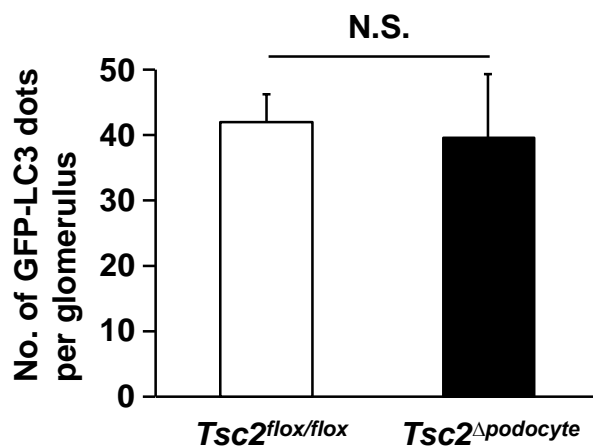

Supplement: S5 Fig — (A) Expression of 858 genes were significantly different in Tsc2Δpodocyte, and 810 out of 858 genes were normalized in rapamycin-treated Tsc2Δpodocyte mice. Rapamaycin-treatment also caused disturbed expression of 76 genes in Rapa- Tsc2Δpodocyte mice, although those levels were similar both in Tsc2Δpodocyte and Tsc2flox/flox mice. (B) Primary cultured podocytes were isolated from Tsc2Δpodocyte mice 1 week after rapamycin treatment, followed by western blot analyses of LC3B type II, p62 and phospho-ULK1 (Ser757). The arrow indicates the band corresponding to LC3B type II. β-tubulin served as the internal control. (C) The graph bars show the number of GFP-LC3 puncta in each glomerulus from Tsc2flox/flox- and Tsc2Δpodocyte-GFP-LC3 transgenic mice. The results are expressed as the mean ± s.d. N.S., not statistically significant. (PDF) [file pone.0229397.s005.pdf]
