# Supplementary material for: Podocyte-specific deletion of tubular sclerosis complex 2 promotes focal segmental glomerulosclerosis and progressive renal failure
Source: PLoS One. 2020 Mar 19;15(3):e0229397. doi: 10.1371/journal.pone.0229397 (PMC7082048; doi:10.1371/journal.pone.0229397)

Full unedited gels for Fig 1A

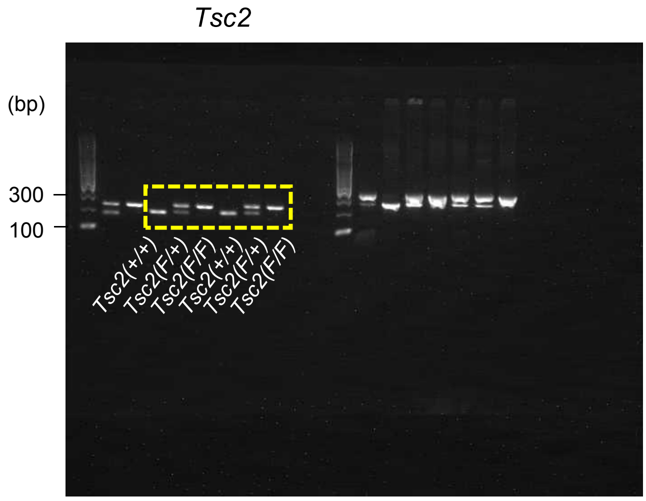

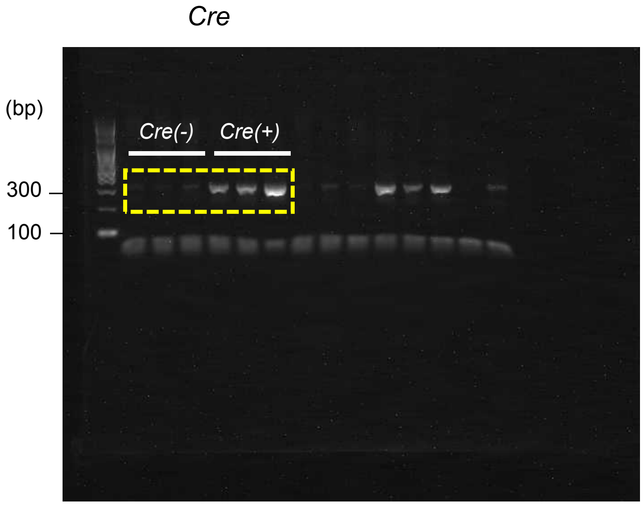

Full unedited gels for Fig 1C

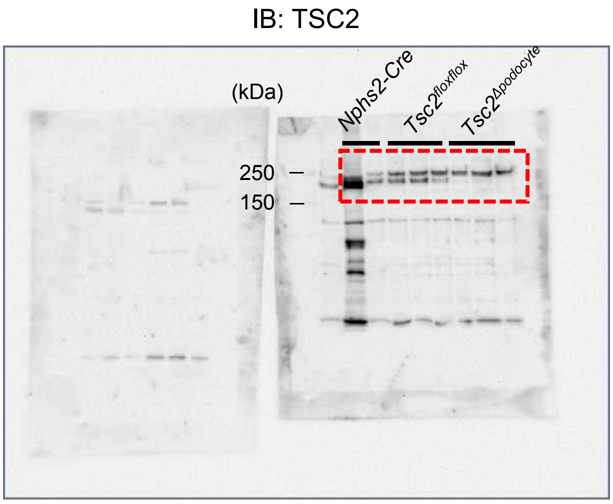

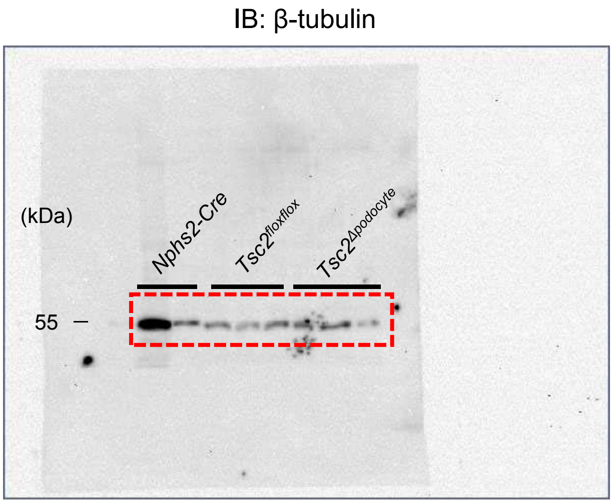

Full unedited gels for Fig 2B

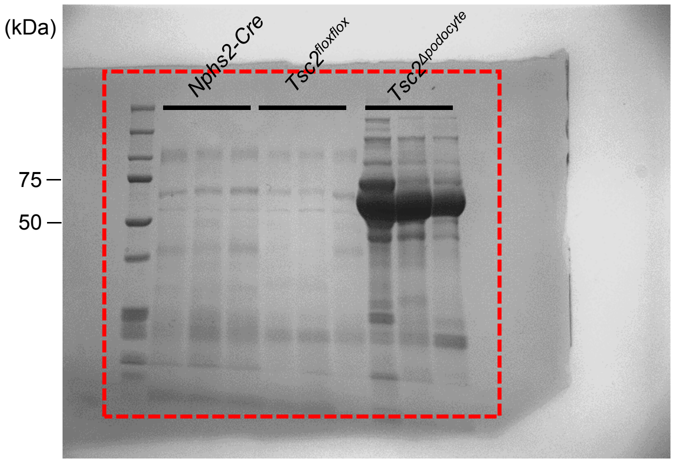

Full unedited gels for Fig 4C

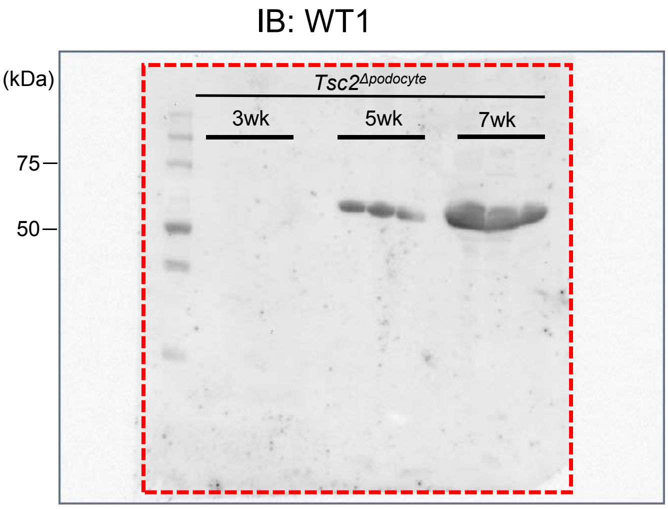

Full unedited gels for Fig 5A

IB: TSC2

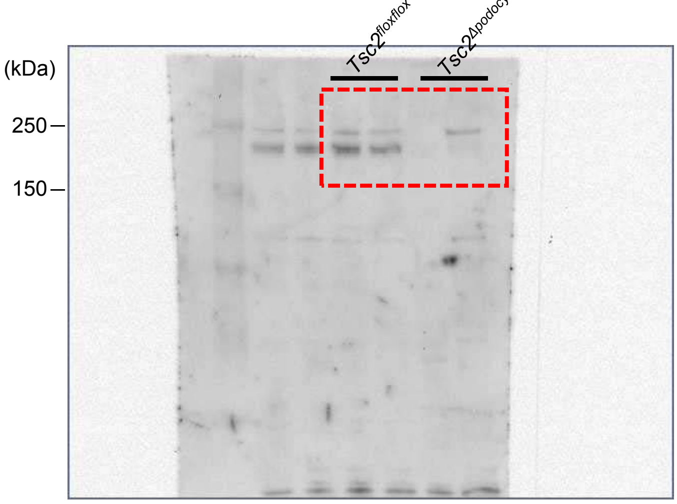

IB: Phospho-4EBP1 (Ser65)

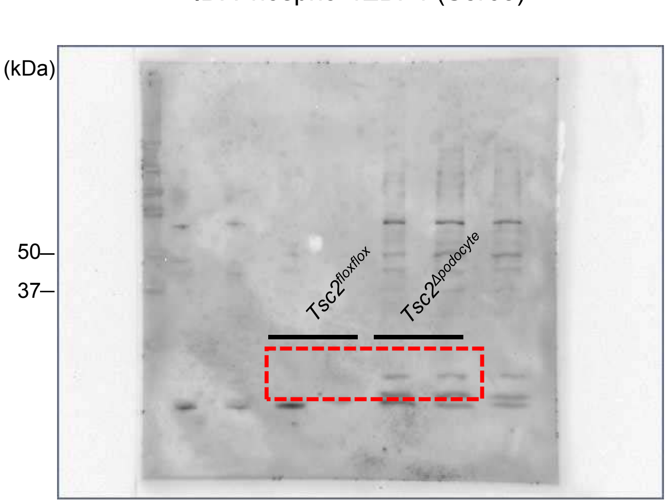

IB: LC3B

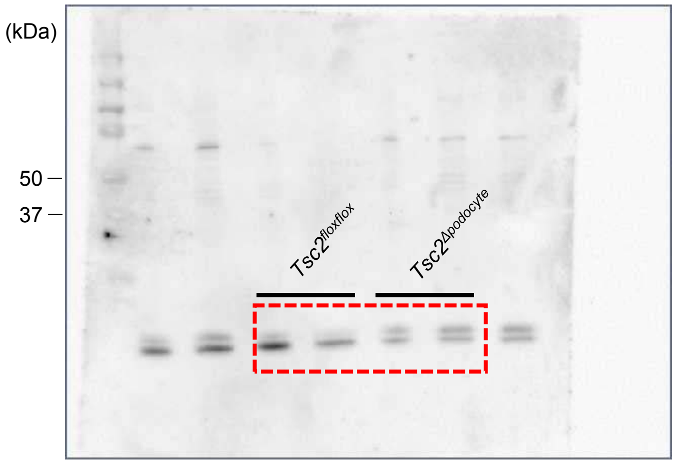

IB: p62

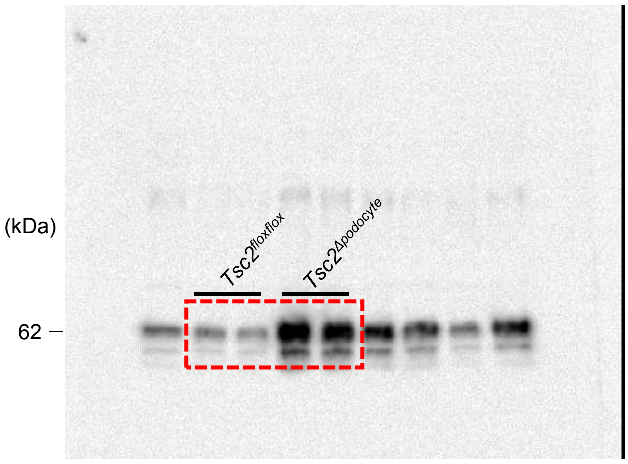

IB: Phospho-ULK1 (Ser757)

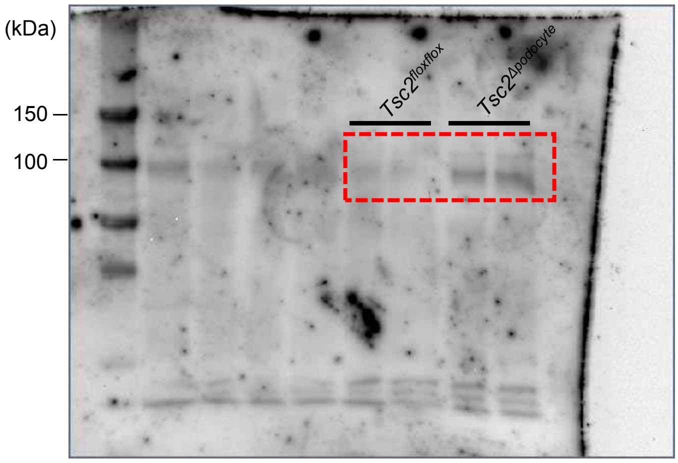

IB: β-tubulin

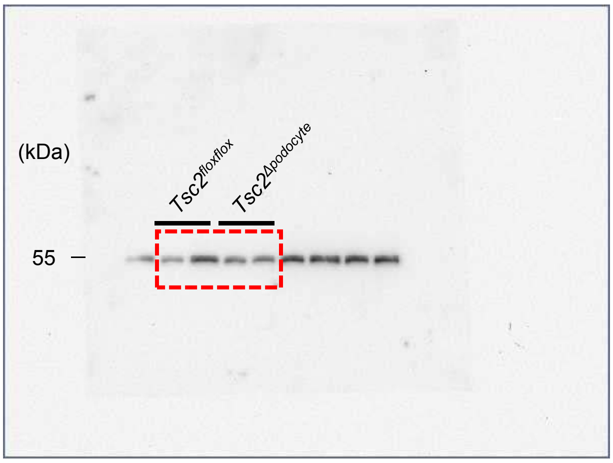

Full unedited gels for Fig 6C

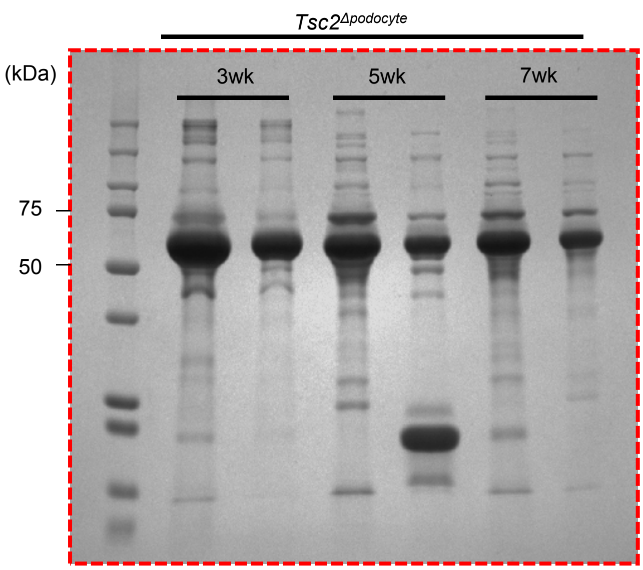

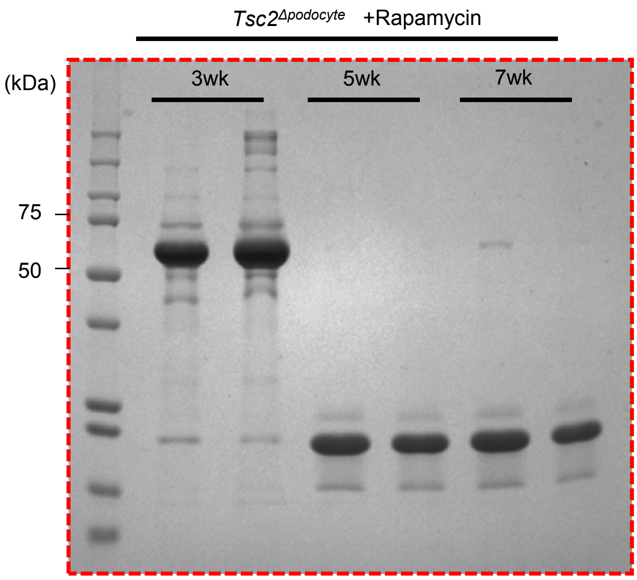

Full unedited gels for Fig 7B

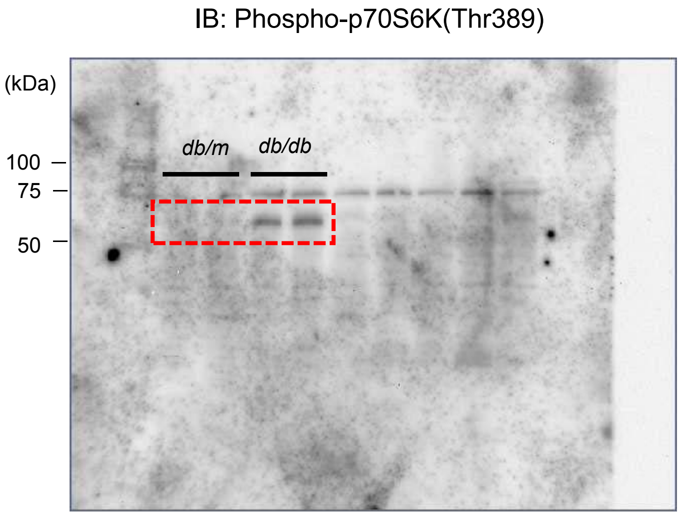

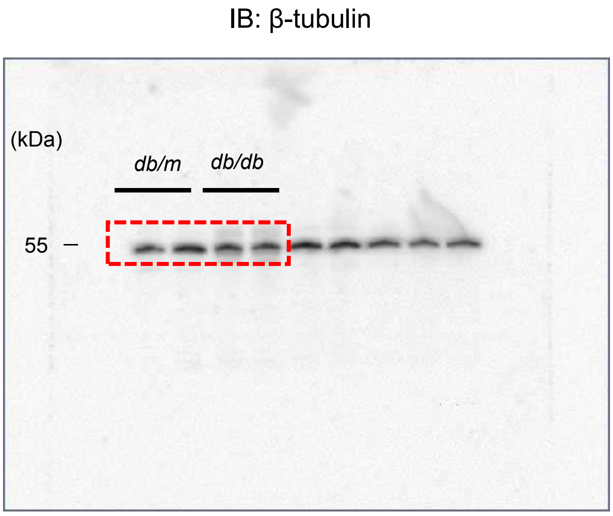

Full unedited gels for S5B Fig.

IB: LC3B

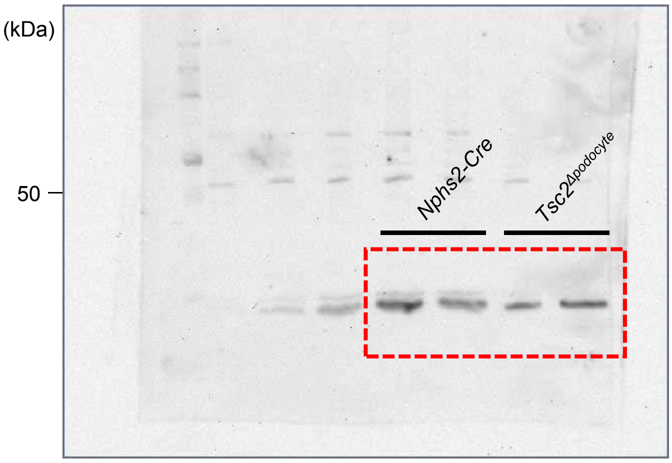

IB: p62

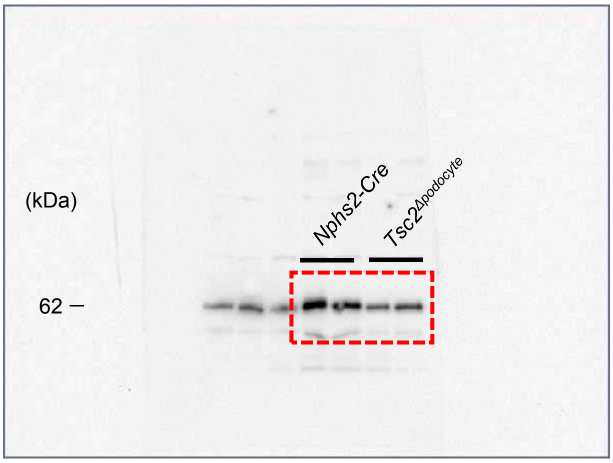

IB: Phospho-ULK1 (Ser757)

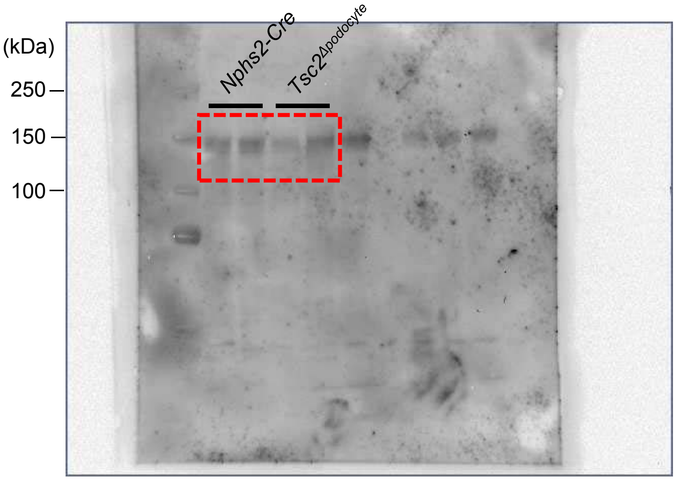

IB: β-tubulin

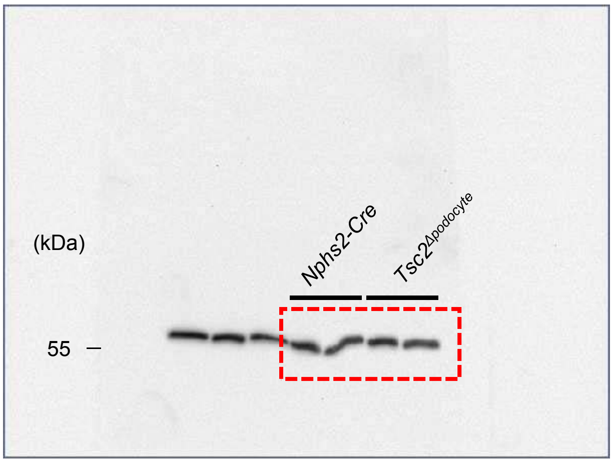

Supplement: S1 Raw images — (PDF) [file pone.0229397.s006.pdf]
