# Supplementary material for: Podocyte-specific deletion of tubular sclerosis complex 2 promotes focal segmental glomerulosclerosis and progressive renal failure
Source: PLoS One. 2020 Mar 19;15(3):e0229397. doi: 10.1371/journal.pone.0229397 (PMC7082048; doi:10.1371/journal.pone.0229397)
Supplement: S1 Table — There were no mice that were euthanized before reaching the experimental endpoint. The numbers of mice that died without humane intervention and euthanized after reaching the experimental endpoint were also summarized. (PDF) [file pone.0229397.s007.pdf]

S1 Table. Overview of the number of mice used in survival analyses. There were no mice that were euthanized before reaching the experimental endpoint. The numbers of mice that died without humane intervention and euthanized after reaching the experimental endpoint were also summarized.

Treatment		*Nphs2-Cre*	*Tsc2* $^{flox/flox}$	*Tsc2* $^{\Delta\ podocyte}$
None (in Fig. 2)	Number of analyzed mice	9	10	32
	Mice that died without humane intervention	0	2	25
	Mice that were euthanized after reaching the experimental endpoint	0	0	7
Saline (in Fig. 6)	Number of analyzed mice	7	9	26
	Mice that died without humane intervention	0	0	21
	Mice that were euthanized after reaching the experimental endpoint	0	0	5
Rapamycin (in Fig. 6)	Number of analyzed mice	17	16	8
	Mice that died without humane intervention	1	2	7
	Mice that were euthanized after reaching the experimental endpoint	0	0	1
