# Supplementary material for: Podocyte-specific deletion of tubular sclerosis complex 2 promotes focal segmental glomerulosclerosis and progressive renal failure
Source: PLoS One. 2020 Mar 19;15(3):e0229397. doi: 10.1371/journal.pone.0229397 (PMC7082048; doi:10.1371/journal.pone.0229397)
Supplement: S2 Table — Data are expressed as mean ± SD (n = 10). Analysis of variance was used between groups; and multiple testing corrections were performed using the Tukey’s method. ACR, urine albumin to creatinin; BUN, blood urea nitrogen; Cre, creatinine; TP, total protein; ALB, alubumin; TC, total cholesterol; TG, triglyceride; HDL-c, high density lipoprotein-cholesterol. aP < 0.05 vs. Nphs2-Cre, bP < 0.05 vs. Tsc2flox/flox. (PDF) [file pone.0229397.s008.pdf]

S2 Table. Characteristics of *Nphs2-Cre*, *Tsc2*$^{flox/flox}$ and *Tsc2*$^{\Delta podocyte}$ mice.

	3 week-old			5 week-old			7 week-old		
	Nphs2-Cre	*Tsc2*$^{flox/flox}$	*Tsc2*$^{\Delta podocyte}$	*Nphs2-Cre*	*Tsc2*$^{flox/flox}$	*Tsc2*$^{\Delta podocyte}$	*Nphs2-Cre*	*Tsc2*$^{flox/flox}$	*Tsc2*$^{\Delta podocyte}$
**Male mice**									
Body weight (g)	19.5 ± 5.5	14.2 ± 5.4	15.4 ± 5.0	26.3 ± 3.0	28.8 ± 3.1	24.4 ± 4.9 [b]	34.7 ± 4.1	31.3 ± 2.7	29.5 ± 5.7 [a]
Fasting blood glucose (mg/dL)	116.9 ± 35.7	109.2 ± 30.7	121.2 ± 31.7	101.2 ± 18.4	132.6 ± 31.3	119.3 ± 24.9	107.8 ± 24.0	127.1 ± 22.2	108.8 ± 27.6
ACR (mg/g Cre)	35.8 ± 8.1	41.9 ± 13.8	608.0 ± 325.1 [a,b]	49.0 ± 18.2	54.6 ± 15.2	873.0 ± 488.0 [a,b]	59.2 ± 27.8	65.0 ± 34.4	764.1 ± 348.7 [a,b]
BUN (mg/dL)	27.1 ± 6.3	30.3 ± 10.2	24.7 ± 4.0	25.3 ± 6.3	25.7 ± 2.8	35.0 ± 12.0 [a,b]	27.5 ± 4.4	26.6 ± 6.7	138.5 ± 172.6 [a]
Serum Cre (mg/dL)	0.18 ± 0.10	0.14 ± 0.10	0.17 ± 0.10	0.19 ± 0.10	0.16 ± 1.00	0.25 ± 0.10	0.29 ± 0.10	0.28 ± 0.10	0.57 ± 0.30 [a,b]
Uric acid (mg/dL)	2.3 ± 1.6	1.3 ± 0.3	1.2 ± 0.5	1.6 ± 1.3	2.3 ± 2.1	1.3 ± 0.3	1.3 ± 0.1	1.7 ± 0.6	2.2 ± 2.0
TP (g/dL)	4.5 ± 0.4	4.2 ± 0.3	4.2 ± 0.4	4.8 ± 0.3	5.1 ± 0.4	4.6 ± 0.3 [b]	5.3 ± 0.4	5.4 ± 0.6	4.7 ± 0.6 [a,b]
ALB (g/dL)	2.5 ± 0.3	2.4 ± 0.3	2.3 ± 0.2	2.5 ± 0.2	2.6 ± 0.2	2.3 ± 0.2 [b]	2.5 ± 0.2	2.5 ± 0.2	1.7 ± 0.4 [a,b]
TC (mg/dL)	106.4 ± 35.0	93.6 ± 16.4	106.6 ± 26.6	134.5 ± 14.7	119.2 ± 8.9	157.0 ± 45.8 [b]	136.6 ± 24.8	116.8 ± 15.5	330.5 ± 145.3 [a,b]
TG (mg/dL)	97.9 ± 28.3	81.7 ± 31.5	89.4 ± 30.0	121.6 ± 35.7	96.4 ± 27.1	105.8 ± 44.0	112.4 ± 31.0	98.6 ± 42.3	186.2 ± 112.1 [b]
HDL-c (mg/dL)	49.0 ± 22.5	31.2 ± 7.1	43.3 ± 17.6	57.7 ± 12.3	47.8 ± 7.8	66.1 ± 23.9	73.1 ± 23.9	57.0 ± 12.8	120.9 ± 64.0 [a,b]
Na (mmol/L)	145.7 ± 3.9	143.9 ± 4.1	145.6 ± 2.2	144.4 ± 2.8	146.8 ± 2.7	142.7 ± 3.5 [b]	143.6 ± 1.8	146.6 ± 2.2	143.8 ± 6.9
K (mmol/L)	3.9 ± 0.6	4.1 ± 0.4	4.1 ± 0.4	4.6 ± 0.8	4.7 ± 0.6	4.6 ± 0.5	4.8 ± 0.5	4.8 ± 0.6	6.0 ± 1.7 [a]
Cl (mmol/L)	123.6 ± 5.7	116.1 ± 3.5	118.1 ± 5.5	122.4 ± 3.4	122.7 ± 2.6	121.4 ± 6.9	123.7 ± 2.4	124.5 ± 5.3	124.3 ± 9.4
**Female mice**									
Body weight (g)	16.7 ± 4.4	15.0 ± 6.1	12.5 ± 3.1	24.6 ± 4.0	19.8 ± 1.7 [a]	21.8 ± 2.6	24.9 ± 2.7	23.6 ± 2.2	22.9 ± 4.1
Fasting blood glucose (mg/dL)	118.5 ± 23.1	98.9 ± 27.8	110.5 ± 37.3	78.2 ± 10.2	73.9 ± 11.0	93.5 ± 19.8 [b]	82.9 ± 17.2	95.3 ± 29.0	104.4 ± 30.6
ACR (mg/g Cre)	40.8 ± 12.0	40.6 ± 31.3	464.1 ± 244.1 [a,b]	46.8 ± 14.8	25.4 ± 9.1	515.2 ± 304.3 [a,b]	32.2 ± 16.6	31.1 ± 17.5	531.7 ± 695.3 [a,b]
BUN (mg/dL)	36.2 ± 10.5	33.9 ± 11.1	42.9 ± 14.3	20.7 ± 4.6	24.8 ± 6.3	56.0 ± 63.3	25.3 ± 8.8	21.0 ± 2.7	158.9 ± 120.7 [a,b]
Serum Cre (mg/dL)	0.19 ± 0.09	0.14 ± 0.05	0.13 ± 0.04 [a]	0.19 ± 0.09	0.15 ± 0.07	0.26 ± 0.08 [a,b]	0.23 ± 0.08	0.23 ± 0.09	0.55 ± 0.30
Uric acid (mg/dL)	1.3 ± 0.3	1.4 ± 0.3	1.3 ± 0.4	1.26 ± 0.3	1.8 ± 1.0	1.3 ± 0.4	1.7 ± 0.6	1.6 ± 0.3	1.5 ± 1.9
TP (g/dL)	4.4 ± 0.7	4.5 ± 0.4	3.9 ± 0.3 [b]	4.72 ± 0.4	4.9 ± 0.5	4.4 ± 0.2 [b]	4.9 ± 0.4	5.2 ± 0.3	4.8 ± 0.8
ALB (g/dL)	2.5 ± 0.3	2.5 ± 0.2	2.1 ± 0.3 [a,b]	2.58 ± 0.2	2.7 ± 0.3	2.2 ± 0.3 [a,b]	2.6 ± 0.2	2.9 ± 0.1	1.5 ± 0.3 [a,b]
TC (mg/dL)	88.8 ± 15.4	82.9 ± 8.3	100.4 ± 60.2	94.5 ± 15.4	88.4 ± 9.3	148.5 ± 55.3 [a,b]	91.9 ± 12.8	92.4 ± 8.8	346.0 ± 116.6 [a,b]
TG (mg/dL)	51.8 ± 16.0	64.6 ± 21.7	53.9 ± 26.7	110.2 ± 21.0	104.6 ± 31.6	100.3 ± 30.6	120.3 ± 36.6	109.0 ± 25.1	185.7 ± 117.0
HDL-c (mg/dL)	30.5 ± 8.6	26.3 ± 4.4	36.3 ± 27.4	35.6 ± 14.3	27.3 ± 3.0	71.6 ± 36.1 [a,b]	36.1 ± 7.0	29.9 ± 5.5	148.8 ± 43.6 [a,b]
Na (mmol/L)	144.7 ± 2.6	143.1 ± 4.3	142.5 ± 3.1	143.4 ± 3.7	143.3 ± 2.8	145.8 ± 2.7	145.3 ± 2.4	143.5 ± 4.1	142.9 ± 6.5
K (mmol/L)	4.3 ± 0.5	4.7 ± 0.7	4.4 ± 0.5	4.07 ± 0.5	4.6 ± 0.6	4.5 ± 0.4	4.4 ± 0.7	4.7 ± 0.6	5.6 ± 1.0 [a,b]
Cl (mmol/L)	118.1 ± 4.8	117.6 ± 4.5	116.6 ± 6.4	121.6 ± 5.7	117.8 ± 4.0	121.9 ± 5.2	120.4 ± 4.4	119.9 ± 1.7	122.4 ± 8.0

Data are expressed as mean ± SD (n =10). Analysis of variance was used between groups; and multiple testing corrections were performed using the Tukey's method. ACR, urine albumin to creatinin; BUN, blood urea nitrogen; Cre, creatinine; TP, total protein; ALB, alubumin; TC, total cholesterol; TG, triglyceride; HDL-c, high density lipoprotein-cholesterol. $^{a}P < 0.05$ *vs. Nphs2-Cre*, $^{b}P < 0.05$ *vs. Tsc2*$^{flox/flox}$.
