# Supplementary material for: Podocyte-specific deletion of tubular sclerosis complex 2 promotes focal segmental glomerulosclerosis and progressive renal failure
Source: PLoS One. 2020 Mar 19;15(3):e0229397. doi: 10.1371/journal.pone.0229397 (PMC7082048; doi:10.1371/journal.pone.0229397)
Supplement: S3 Table — Data are expressed as mean ± SD (n = 5). Analysis of variance was used between groups; and multiple testing corrections were performed using the Tukey’s method. There were no significant differences in the biochemical parameters among rapamycin-treated Nphs2-Cre, Tsc2flox/flox and Tsc2Δpodocyte mice. Abbreviations are as in S2 Table. (PDF) [file pone.0229397.s009.pdf]

S3 Table. Characteristics of rapamycin-treated *Nphs2-Cre, $Tsc2^{flox/flox}$* and *$Tsc2^{\Delta podocyte}$* mice.

	3 week-old			5 week-old			7 week-old			11 week-old		
	Nphs2-Cre	*$Tsc2^{flox/flox}$*	*$Tsc2^{\Delta\ podocyte}$*	*Nphs2-Cre*	*$Tsc2^{flox/flox}$*	*$Tsc2^{\Delta podocyte}$*	*Nphs2-Cre*	*$Tsc2^{flox/flox}$*	Tsc2Δpodocyte	*Nphs2-Cre*	*$Tsc2^{flox/flox}$*	*$Tsc2^{\Delta podocyte}$*
Body weight (g)	12.2 ± 1.3	13.8 ± 1.1	13.2 ± 2.4	26.7 ± 2.3	23.5 ± 5.0	22.0 ± 3.5	31.3 ± 2.4	22.9 ± 4.4	24.9 ± 2.1	32.8 ± 2.4	25.7 ± 5.2	26.9 ± 3.0
Fasting blood glucose (mg/dL)	104.0 ± 22.7	86.6 ± 23.9	88.4 ± 20.4	85.5 ± 14.0	105.4 ± 23.2	103.2 ± 21.6	94.3 ± 16.3	108.8 ± 23.8	135.6 ± 35.4	100.6 ± 19.2	99.2 ± 12.2	105.7 ± 99.2
BUN (mg/dL)	26.4 ± 3.2	27.7 ± 11.7	33.4 ± 8.4	21.8 ± 2.3	19.8 ± 3.9	19.6 ± 4.2	24.3 ± 7.4	16.3 ± 3.0	21.6 ± 4.2	18.9 ± 3.6	18.1 ± 4.9	24.7 ± 7.6
Serum Cre (mg/dL)	0.10 ± 0.03	0.09 ± 0.02	0.09 ± 0.02	0.13 ± 0.05	0.18 ± 0.10	0.16 ± 0.08	0.24 ± 0.17	0.20 ± 0.16	0.12 ± 0.04	0.36 ± 0.24	0.21 ± 0.12	0.18 ± 0.11
TC (mg/dL)	84.6 ± 33.9	103.4 ± 23.0	105.6 ± 26.3	165.8 ± 9.4	122.4 ± 28.4	149.0 ± 16.0	176.6 ± 18.3	131.2 ± 34.1	162.0 ± 41.6	160.8 ± 27.8	120.4 ± 24.2	157.3 ± 21.1
TG (mg/dL)	57.0 ± 15.0	60.0 ± 25.7	89.8 ± 54.5	154.8 ± 14.2	134.4 ± 16.6	126.6 ± 25.9	104.6 ± 13.6	72.0 ± 15.4	75.4 ± 15.9	84.8 ± 14.0	71.0 ± 11.7	73.8 ± 51.7
HDL-c (mg/dL)	30.4 ± 16.7	51.4 ± 17.1	41.8 ± 19.1	89.0 ± 6.0	53.4 ± 17.8	75.0 ± 13.5	91.6 ± 13.0	61.0 ± 24.0	85.2 ± 19.8	86.4 ± 10.4	55.6 ± 20.0	76.5 ± 9.3
Na (mmol/L)	151.4 ± 2.9	150.8 ± 3.9	151.2 ± 3.3	151.0 ± 2.4	149.4 ± 3.0	151.0 ± 3.1	154.8 ± 3.4	154.2 ± 3.8	150.8 ± 1.8	151.4 ± 3.4	152.2 ± 2.5	150.8 ± 1.0
K (mmol/L)	4.8 ± 0.8	4.5 ± 1.0	5.0 ± 0.7	4.5 ± 0.1	4.6 ± 0.7	4.4 ± 0.6	5.1 ± 0.4	4.4 ± 0.1	4.4 ± 0.8	5.5 ± 0.2	5.2 ± 0.7	6.0 ± 0.8
Cl (mmol/L)	121.4 ± 3.8	121.6 ± 2.5	120.6 ± 3.3	127.6 ± 2.3	125.6 ± 4.0	126.6 ± 2.5	134.2 ± 1.9	126.8 ± 3.2	125.4 ± 3.0	125.4 ± 3.4	125.0 ± 2.9	123.8 ± 3.1

Data are expressed as mean ± SD (n = 5). Analysis of variance was used between groups; and multiple testing corrections were performed using the Tukey's method. There were no significant differences in the biochemical parameters among rapamycin-treated *Nph2-Cre, $Tsc2^{flox/flox}$* and *$Tsc2^{\Delta\ podocyte}$* mice. Abbreviations are as in S2 Table.
