# Supplementary material for: Podocyte-specific deletion of tubular sclerosis complex 2 promotes focal segmental glomerulosclerosis and progressive renal failure
Source: PLoS One. 2020 Mar 19;15(3):e0229397. doi: 10.1371/journal.pone.0229397 (PMC7082048; doi:10.1371/journal.pone.0229397)
Supplement: S4 Table — (PDF) [file pone.0229397.s010.pdf]

S4 Table. Clinical characteristics of normal control subjects and patients diagnosed with FSGS perihilar variant

Disease group	Patient No.	Gender	Age (years by decade)	Body mass index (kg/m^2)	SBP / DBP (mmHg)	HbA1c (%)	Serum Creatinine (mg/dL)	eGFR (ml/min/1.73m^2)	Urine protein to creatinin ratio (g/gCre)	Number of pS6(+)-glomeruli / total number of glomeruli
Normal control	1	F	41 — 50	20.8	124 / 84	5.4	1.24	37	7.40	0 / 10
Normal control	2	F	61 — 70	24.4	119 / 66	5.6	0.80	55	0.11	7 / 18
Normal control	3	F	21 — 30	20.9	115 / 74	5.2	0.80	101	0.19	11 / 26
FSGS (perihilar variant)	1	M	21 — 30	33.6	114 / 56	5.6	1.01	72	0.65	18 / 18
FSGS (perihilar variant)	2	M	41 — 50	30.9	114 / 76	6.1	0.88	74	0.95	7 / 10
FSGS (perihilar variant)	3	M	31 — 40	25.3	124 / 78	5.4	0.86	81	3.30	11 / 15
